# Urbanization can increase the invasive potential of alien species

**DOI:** 10.1111/1365-2656.13293

**Published:** 2020-07-28

**Authors:** Piatã Santana Marques, Luisa Resende Manna, Therese Clara Frauendorf, Eugenia Zandonà, Rosana Mazzoni, Rana El‐Sabaawi

**Affiliations:** ^1^ Biology Department University of Victoria Victoria BC Canada; ^2^ Departamento de Ecologia Universidade do Estado do Rio de Janeiro Rio de Janeiro Brazil

**Keywords:** diet, intraspecific traits, invasiveness, tropics, urban biodiversity, urban ecology, urban stream

## Abstract

1. Alien species often flourish and become invasive in urban ecosystems. How and why invaders succeed in urban systems is an important, yet poorly understood, question. We investigate whether the success of urban invaders is related to changes in species traits that enhance invasive potential. We also explore whether a trophic mechanism helps explain the success of invaders in urban systems. We use the guppy *Poecilia reticulata*, a globally distributed alien species that has invaded both urban and non‐urban systems, as our model.
2. We first characterize the effect of urbanization on streams where guppies are present. We measure guppy invasion success using their population density and size‐frequency. Then we assess how traits that are related to the potential of guppies to invade (life history and condition) respond to urbanization. Next, we explore how urbanization affects the availability of food for guppies and their diets. We also test if the presence of other fish species grants biological resistance to invasion by dampening guppy invasive potential.
3. We find that urban streams have high concentrations of ammonium and faecal coliforms, indicating contamination from sewage. On average, guppy populations from urban streams have 26× higher density and larger body sizes than non‐urban populations. Urban guppies are in better condition and have on average five more offspring than non‐urban guppies. Urbanization increases the availability and consumption of highly nutritious food (chironomid larvae) by guppies. We find a positive relationship between the consumption of chironomids and both fecundity and condition. The presence of other fish species in urban streams often has a negative but small effect on guppy traits and density.
4. Our data suggest a relaxation of trade‐offs that shape life‐history traits which is related to increased food resources in urban streams. These indicate that urbanization enhances the invasive potential of guppies through a trophic mechanism that simultaneously increases reproduction and somatic investment. Such mechanism is likely widespread because chironomids are often highly abundant in urban systems. Thus, not only guppies but also other invasive species can take advantage of such a resource to invest in traits that enhance invasion success.

## Graphical abstract

This paper looks at how urbanization enhances the success of invaders by changing species traits. The authors find that urbanization provides high food quality and quantity, which allows invasive guppies to increase body size and condition, number of offspring and population density. This suggests that urbanization promotes a highly invasive phenotype.

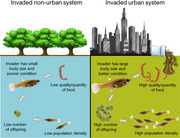

## 1 INTRODUCTION

Disturbances, associated with human activities such as urbanization, can transform ecosystems in dramatic ways. Urbanization replaces green spaces with impervious surfaces and built structures and increases pollution. This reduces biodiversity, creating a highly simplified community where specialized sensitive taxa decline, while generalist tolerant taxa flourish (Faeth, Bang, & Saari, 2011). The movement of people and goods also provides opportunities for organisms to be transported among different cities (Wilson, Dormontt, Prentis, Lowe, & Richardson, 2009). As a result, urban ecosystems contain a large number of alien species, defined as non‐native species whose presence in a region results from human actions (Lososová, Chytrý, Danihelka, Tichý, & Ricotta, 2016; Richardson, Pysek, & Carlton, 2011).

Alien species become invasive when they maintain large self‐replacing populations over many life cycles far from the site of introduction (Richardson et al., 2011). Many invasive species are successful in urban environments, as seen by their very high densities, with a range of negative consequences for humans and ecosystems (Haag‐Wackernagell & Moch, 2004; Shochat et al., 2010). The diversity of competitors and predators can provide biotic resistance against invasion (Levine & D'Antonio, 1999), but evidence for this putative effect in urban systems is lacking.

Although the occurrence of invasive species in cities has been reported for many decades, there is a poor understanding of the mechanisms facilitating invasion in urban areas (Cadotte, Yasui, Livingstone, & MacIvor, 2017). It has been suggested that invasive species are successful in urban systems because they happen to have traits [i.e. characteristic measured at the individual level which affect fitness, sensu Violle et al. (2007)] that are advantageous in urban systems (Byers, 2002). For example, plants with fleshy fruits and birds with low fear of humans are thought to have higher success invading urban systems (Aronson, Handel, & Clemants, 2007; Carrete & Tella, 2011). However, urbanization also represents a strong selective force for trait changes (Alberti et al., 2017). Thus, it is possible that the success of urban invaders is also related to changes to existing traits that increase their potential to invade ecosystems.

The invasive potential of a species is closely related to its life‐history traits. Organisms with ‘rapid’ life‐history traits, such as fast growth (which lead to greater body sizes) and high fecundity, are anticipated to have higher invasive potential because such traits increase the chances of invasion success (Allen, Street, & Capellini, 2017; Capellini, Baker, Allen, Street, & Venditti, 2015). Current life‐history theory proposes that life‐history traits are shaped, in most part, by nutritional trade‐offs because individuals have limited nutritional resources to allocate into multiple tasks such as somatic growth (e.g. body size), reproduction (e.g. number of offspring) and navigating biotic interactions (i.e. competition and predator avoidance; Roff, 1992; Stearns, 1992). However, urbanization can increase the availability of food resources in terrestrial and aquatic ecosystems, potentially relaxing nutritional trade‐offs that shape life‐history traits (Snell‐Rood et al., 2015). This might allow organisms to increase their investment in multiple traits simultaneously, towards faster life histories with enhanced invasive potential (Shik & Dussutour, 2020). However, the extent to which the success of invasive species in urban systems is related to changes to traits that enhance invasive potential and the ecological mechanisms facilitating urban invasion are poorly known.

In this study we assess whether urban disturbance sharpens the invasive potential and increases the success of invasive species. Our overarching hypothesis is that invasive species take advantage of the increased food resource in urban systems to invest in traits that enhance their success and invasive potential (i.e. invasiveness sensu Richardson et al., 2011). We test this using a global invader, the guppy *Poecilia reticulata*. Guppies are a valuable model because they have been used for decades in preserved, non‐urban systems for testing how changes in the environment affect traits, specially life‐history traits, as predicted by classic theories (Reznick, Butler IV, & Rodd, 2001; Reznick & Endler, 1982; Travis et al., 2014).

We begin by comparing environmental metrics between urban and non‐urban streams in order to characterize the effects of urbanization to streams. We predict that urban guppy populations, as seen for other species, are more successful by attaining higher density and increased body sizes than non‐urban populations (Schröder, Nilsson, Persson, Kooten, & Reichstein, 2009; Shochat et al., 2010). We then compare life‐history traits of urban and non‐urban guppies because such traits affect invasiveness in many different species (Richardson et al., 2011). We hypothesize that urbanization increases guppy invasiveness by enhancing traits related to reproduction and somatic growth. Then, we test if urbanization increases the availability and the consumption of food by guppies, especially chironomids (non‐biting midge larvae).

Urbanization is expected to increase the biomass of invertebrates in urban streams, particularly that of tolerant taxa such as Chironomidae (Yule, Gan, Jinggut, & Lee, 2015). Chironomids are highly nutritious (have high nitrogen concentration per body mass), and are consumed by different urban species, including guppies (Ganassin, Frota, Muniz, Baumgartner, & Hahn, 2019; Mackintosh, Davis, & Thompson, 2017). The consumption of nutritious food has been suggested to affect life‐history traits across species (Swanson et al., 2016). We predict that improved nutrition in urban streams allows guppies to invest simultaneously towards somatic growth and reproduction, providing evidence for a relaxation of life‐history trade‐offs. Finally, we investigate whether fish biodiversity (the presence of potential fish competitors and predators) affect guppy invasion. Biodiversity is thought to enhance biotic resistance to invasive species (Levine & D'Antonio, 1999). We therefore predict that fish biodiversity will reduce guppy density, body size, investment in reproduction and condition.

## 2 MATERIALS AND METHODS

Our study took place in Rio de Janeiro, Brazil, where guppies have invaded many brackish and freshwater systems since at least 1942, when the first guppy specimens were catalogued by the National Museum (MNRJ 3646; Rocha, Bergallo, & Mazzoni, 2011). We selected six urban stream reaches and six non‐urban stream reaches, with the selection of urban streams based on the presence of development and evident direct sewage input, while non‐urban streams were located inside parks and forest reserves. Most reaches were located in small first to second order streams in different watersheds (Figure S1). Two urban reaches were located downstream of the non‐urban reaches in the same stream (ELSU and WPS reaches). In these cases, reaches were separated by natural and artificial barriers forming waterfalls which prevented the upstream movement of fish. Each reach was 30 m in length. Within each treatment (urban, non‐urban), three reaches had guppies as the only fish (guppy only), while in the other three guppies co‐occurred with potential competitors and predators (guppy and other fish species; Table S1). When guppies co‐occurred with other fishes, the total number of fish species ranged from 3 to 10. Each reach had at least one potential predator, the catfish *Rhamdia quelen* (Bonato, Delariva, & Silva, 2012) and one potential omnivorous competitor such as the poecilid *Phalloceros* sp. or the pearl cichlid *Geophagus brasiliensi*s (Bastos, Condini, Varela Junior, & Garcia, 2011; Deus & Petrere‐Junior, 2003). Whenever possible we sampled each reach in 2 years (2016–2017).

### 2.1 Environmental variables

At each reach, we measured pH, conductivity (Spec µS/cm), temperature (°C), dissolved oxygen (mg/L) and % canopy (details in Table S2). Ammonium (NH_4_) is an important limiting nutrient in aquatic ecosystems, that can be originated from various sources including sewage (Bernhardt, Band, Walsh, & Berke, 2008). We collected and filtered five water samples along each reach and analysed them for NH_4_ concentration (µg/L) using a fluorometric method (Holmes, Aminot, Kérouel, Hooker, & Peterson, 1999). We also took a composite sample, composed of three subsamples, of water to estimate faecal coliforms (*Escherichia coli* counts, FC), which indicate contamination with human faeces (Edberg, Rice, Karlin, & Allen, 2000). The samples were refrigerated (7°C) and sent (within 24 hr of collection) to a private analytical laboratory where the most probable number of *E*. *coli* cells (MPN/100 ml) was estimated following a dilution method (Rice, Baird, Eaton, & Clesceri, 2012).

### 2.2 Population density, size structure and fish biodiversity

We estimated guppy density following a depletion method (Carle & Strub, 1978). Each stream reach (~30 m in length) was blocked with fine mesh nets and electrofished three consecutive times using a backpack electrofisher (LR‐24 Smith‐Root^®^). The difference in the number of guppies captured (both male and females) in each of the three fishing events allowed us to estimate guppy density (GD, ind/m^2^), using the fsa package for r (Ogle, Wheeler, & Dino, 2018; R Core Team, 2018). A total of 3,224 female guppies (411 ± 84 urban and 126 ± 39 non‐urban individuals per reach) were either measured for standard length to the nearest millimetre (SL, mm) and returned to the stream, or euthanized with a high dose of anaesthetic (Tricaine, MS‐222), fixed in formalin (10%) and brought to the laboratory for standard length measurements and further analyses. Guppies were collected and euthanized following protocols approved by the University of Victoria (2016‐008) and the State University of Rio de Janeiro (UERJ CEUA/005/2016) animal care committees, as well as the Brazilian Ministry for the Environment (IBAMA 16152‐1). We used body length to build cumulative length distributions in order to determine the size class distribution of the guppies in each reach (Neumann & Allen, 2007). All co‐occurring fish species were identified to estimate fish biodiversity.

### 2.3 Guppy reproductive traits and condition

From the euthanized female guppies, a total of 494 urban (82 ± 12 per reach) and 241 non‐urban (40 ± 12 per reach) were used for assessing life‐history traits. Female guppies are viviparous, they store sperm, self‐fertilize their eggs and reproduce continuously by giving birth to a new batch of offspring every 3 months on average (Magurran, 2005). We selected only mature females [having mature eggs, sensu Haynes (1995), or embryos] for the life‐history study. For each female, we estimated three reproduction‐related traits as follows: fecundity measured as the number of offspring (number of embryos and mature eggs, NO), the gonad mass (dry mass, mg) and reproductive allotment (gonad dry mass/total body dry mass minus gonads). We assessed the condition (CO) of each female using an hepatosomatic index which was calculated as the ratio of liver dry mass (mg) and eviscerated body dry mass (mg), which provided an estimate of the amount of stored nutrients such as lipids (Dagar, Zilberg, Cohen, Boussiba, & Khozin‐Goldberg, 2010; Lloret, Shulman, & Love, 2014).

### 2.4 Invertebrate availability

In each reach, three randomly placed samples of benthic invertebrates were collected using a surber sampler (area = 0.09 m^2^). For each sample, invertebrate families were identified, measured (mm) and mass‐length regressions from the literature were used to estimate invertebrate biomass (dry mass mg/m^2^; Benke, Huryn, Smock, & Wallace, 1999). Since not all invertebrates can be consumed by guppies because of gape‐size limitations, we only considered taxa found in gut contents and invertebrates smaller than 6 mm (mean size of invertebrates consumed by guppies) to estimate the invertebrate biomass available as food for guppies (IB, mg/m^2^; Figure S2). Because chironomids are an important food source for guppies (Zandonà et al., 2011), we also estimated the biomass of chironomids only (CB, mg/m^2^).

### 2.5 Diet analysis

From the euthanized female guppies, we retained a total of 116 urban (29 ± 3 per reach) and 120 non‐urban (20 ± 3 per reach) for gut content analysis. For each guppy, the foregut was sectioned to the point where the gut turns 180° and the gut contents were analysed following the gridded microscope slide technique (Zandonà et al., 2011). The slide area occupied by invertebrates, algae and detritus (i.e. silt and amorphous material) was estimated (mm^2^). We identified the invertebrates to the lowest taxonomic level possible, generally family, using taxonomic keys (Merritt & Cummins, 1996; Mugnai, Nessimian, & Baptista, 2010). Then, we used the total area of the slide occupied to estimate the proportion of invertebrates, algae and detritus consumed, following: *P_i_* = *A_i_*/*A_t_*, where, *P_i_* = proportion of the food item, *A_i_* = area of the food item, *A_t_* = total area occupied by all diet items. The proportion of invertebrates consumed was further divided into proportion of chironomids (PC) and proportion of other invertebrates, which include insect families within Diptera, Lepidoptera and Trichoptera. Individuals with empty guts were removed from any analysis involving diet.

### 2.6 Statistical analyses

#### 2.6.1 Comparisons between urban and non‐urban systems

We performed a PCA analysis based on the correlation matrix of the normalized environmental variables: pH, conductivity (Spec µS/cm), temperature, dissolved oxygen, % canopy cover, ammonium concentration, faecal coliforms and sampling year (Table S2). The PCA plot includes reaches sampled in both years (2016–2017) separately. This analysis assessed whether urban reaches were more similar to each other than to non‐urban reaches, as indicated by how the reaches clustered in the PCA plot. PCA was performed using the function prcomp and the package factoextra for r (Kassambara & Mundt, 2016). Due to missing data, we estimated DO for JOA reach, FC for CAR and ELSU reaches and NH_4_ for CATO reach based on the mean of the treatment per year (urban, non‐urban; Table S2).

We further compared NH_4_ concentration, faecal coliforms and estimated invertebrate biomass [all invertebrates (IB) and chironomids (CB)] between urban and non‐urban reaches using generalized linear mixed models (GLMMs). We included urbanization (urban vs. non‐urban), year and fish biodiversity (guppy + other fish, guppy only) as fixed factors, and reach identity (i.e. stream reach or site) as a random factor. In analyses of NH_4_, IB and CB, we added guppy density as a fixed factor since it can potentially influence these variables through consumption or excretion (El‐Sabaawi et al., 2015). Reach identity was included to account for any additional environmental differences among stream reaches that were not directly measured in our study.

We tested for changes to the average proportion of chironomids in gut contents between urban and non‐urban guppies using GLMMs. We also tested for differences in the number of offspring, condition, gonad mass and reproductive allotment. In all models, we included urbanization, year, fish biodiversity (guppy + other fish vs. guppy only), body length and guppy density as fixed factors, and reach identity as a random factor.

We used cumulative length distributions to estimate variation in body size between urban and non‐urban guppies under low and high biotic interactions. We tested for differences in length distribution using a bootstrapped Kolmogorov–Smirnov test (K‐S two‐sample test) from the matching package for r which is appropriate for discrete length data (Ogle, 2015; Sekhon, 2011). We also tested for differences in guppy density between urban and non‐urban reaches using GLMMs. In this model we included year and fish biodiversity as fixed factors, and reach identity as random factor.

We fitted all models with the lme4 package for r (Bates, Machler, Bolker, & Walker, 2015). We chose the link function based on the best fit for the data. Model fit was evaluated visually using Q‐Q plots. We tested the models for multicollinearity with the VIF function of the car package for r (Fox & Weisberg, 2011). The variance explained by both fixed plus random factor (conditional *R* square, $R_{c}^{2}$), and the variance explained only by the fixed factors (marginal *R* square, $R_{m}^{2}$) were estimated using the ‘r.squaredGLMM’ function of the r package mumin.

#### 2.6.2 Testing the link between chironomid consumption and guppy traits

We used linear mixed models (LMMs) to explore the relationship between the consumption of chironomids and guppy traits across urban and non‐urban reaches. We built three separate models using the mean for each trait per reach—number of offspring (NO), condition (CO) and body length (SL), as response variables. We used the proportion of chironomids consumed (PC), sampling year (YR), fish biodiversity (guppy + other fish vs. guppy only), and guppy density (GD) as fixed factors, and reach identity (RI) as a random factor in all models. Body length (SL) was included as a fixed factor when modelling condition and the number of offspring. The global models for number of offspring, condition and body length were subjected to model selection based on AICc criteria (Grueber, Nakagawa, Laws, & Jamieson, 2011). Only the best models (ΔAICc < 2) were considered because they have substantial empirical support (Burnham & Anderson, 2002). The best models were retrieved and averaged to estimate the coefficients of the predictor variables (coef_avg_; Grueber et al., 2011). All models were fitted with the lme4 package for r (Bates et al., 2015). Model selection and averaging were performed with the mumin package for r (Bartoń, 2018). We further assessed the link between chironomid consumption and number of offspring, condition and body length using the likelihood ratio test from the ‘lrtest’ function of the lmtest package for r (Hothorn et al., 2018). All plots were built with package ggplot2 for r and deviations reported are standard errors (Wickham, 2009).

## 3 RESULTS

### 3.1 Environmental variables

**Figure 1:**
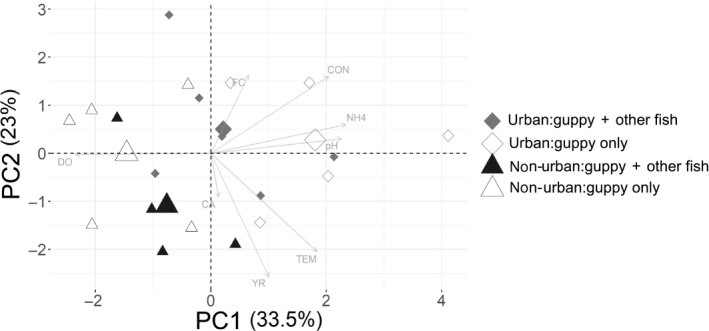
Principal component analysis (PCA) showing urban reaches where guppies co‐occur with other fish species (solid grey diamonds, urban:guppy + other fish), urban reaches where guppies are the only fish species (hollow diamonds, urban:guppy only), non‐urban reaches where guppies co‐occur with other fish species (solid black triangles, non‐urban:guppy + other fish) and non‐urban reaches where guppies are the only fish species (hollow triangles, non‐urban:guppy only). Whenever possible each reach was sampled in two different years (2016 and 2017; Table S2). Symbols show reach per year. Large symbols represent the mean for all the reaches in each condition. The analysis is based on the environmental variables: CON = Specific conductivity (Spec µS/cm), TEM = temperature (°C), DO = dissolved oxygen (mg/L), CA = canopy cover (%), FC = faecal coliforms (*Escherichia coli*, MPN/100 ml), NH4 = ammonium concentration (µg/L) and sampling year (YR)

The PCA showed that urban reaches were environmentally more similar to each other than non‐urban reaches in both years. The PCA explained 56% of the total variation among reaches. PC1 explained 33.5% of variation and was driven by differences in ammonium, dissolved oxygen and pH between urban and non‐urban reaches (Figure 1; Table S3). PC2 explained 23% of the variation and was driven mainly by differences between sampling years (Figure 1; Table S3). However, there was no obvious clustering pattern by year, suggesting that urban effects were consistent across the two sampling years. The variation in abiotic factors between reaches differing in fish biodiversity was also minor.

Generalized linear models confirmed the urban versus non‐urban differences observed in the PCA. Urban reaches had higher mean (±*SE*) ammonium concentrations than non‐urban reaches (2,756 ± 1,150 and 15 ± 3 µg/ml, respectively; GLMM, *t* = 12.5, *p* < 0.001), with a positive effect of guppy density on ammonium concentration (GLMM, *t* = 3.5, *p* < 0.001; Table S4). There were markedly higher concentrations of faecal coliform in urban reaches compared to non‐urban reaches (187,743 ± 8,379 and 1,801 ± 867 MPN/100 ml, respectively; GLMM, *t* = 7.8, *p* < 0.001), with higher *E*. *coli* counts in 2016 than 2017 (136.5 ± 129.6 and 76.3 ± 40.6, respectively; GLMM, *t* = −2.31, *p* = 0.02). Otherwise, there were no significant differences between years or in abiotic conditions between reaches that differed in fish diversity (Table S4).

### 3.2 Guppy success, invasiveness and the effect of biodiversity

**Figure 2:**
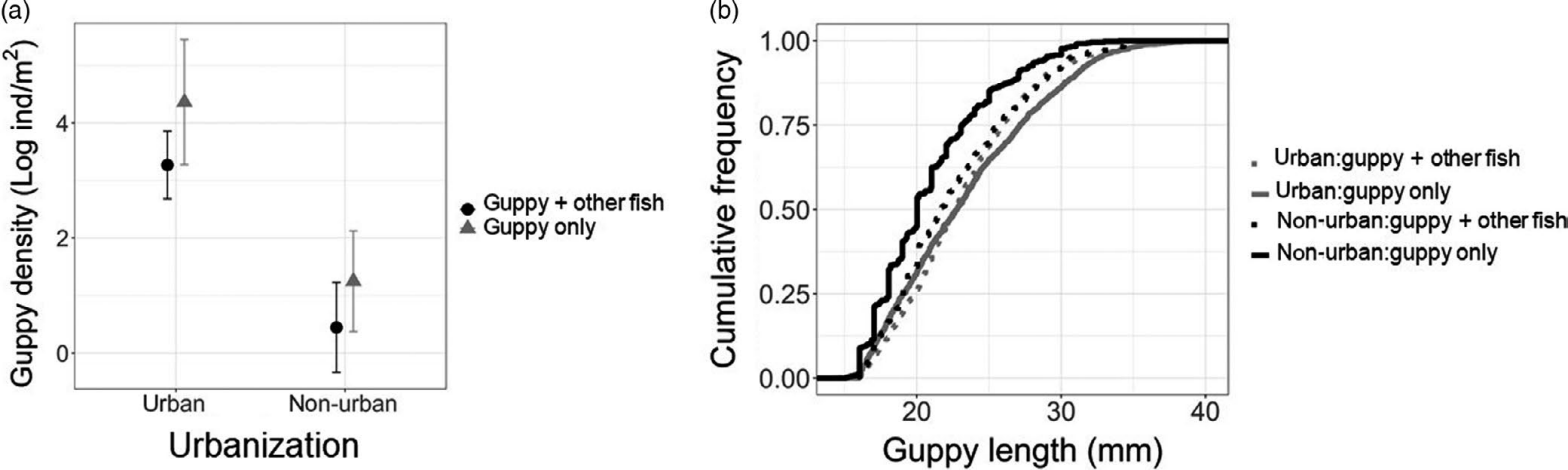
Guppy population metrics. Panel (a) shows guppy density estimated as mean number of individuals, both males and females, per metre square. Bars represent the standard error of the mean. Black circles indicate reaches where guppies co‐occur with other fish species (guppy + other fish) and grey triangles indicate reaches where guppies are the only fish species (guppy only). Panel (b) shows the empirical cumulative distribution function curves for guppy length. Lines represent the proportion of guppies at each length category in three urban reaches where guppies occur with other fish (grey dotted line, urban:guppy + other fish), three urban reaches where guppies are the only fish species (solid grey line, urban:guppy only), three non‐urban reaches where guppies occur with other fishes (black dotted line, non‐urban:guppy + other fish) and three non‐urban reaches where guppies are the only fish species (black solid line, non‐urban:guppy only). Data shown combine both sampling years (2016 and 2017)

Guppy density was ~26× higher in urban than non‐urban in reaches (78 ± 34 and 3 ± 1 ind/m^2^, respectively; GLMM, *t* = 6.7, *p* < 0.001), with a lower overall density in 2016 compared to 2017 (33 ± 16 and 52 ± 35 ind/m^2^, respectively; GLMM, *t* = 2.4, *p* = 0.02) and no effect of biodiversity (Figure 2a; Tables S4 and S5). The cumulative length frequency distribution showed that urban guppies had larger body lengths than non‐urban guppies (K‐S test *p* < 0.001). The presence of other fish species slightly reduced guppy body size but did not entirely remove the effect of urbanization (K‐S test *p* < 0.01; Figure 2b).

**Figure 3:**
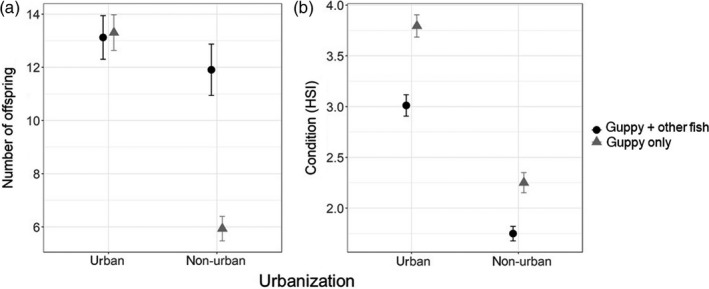
Comparing traits of urban and non‐urban guppies. In both treatments (urban and non‐urban) guppies occur in stream reaches with other fish species (black circles, guppy + other fish) and in reaches where guppies are the only fish species (grey triangles, guppy only). The panels show: (a) the number of offspring, estimated as the mean total counts of embryos and mature eggs for all the females, (b) guppy condition estimated for the female guppies using the mean hepatosomatic index (HSI). Symbols represent the means and bars as the standard error of the mean. Data shown include two sampling years (2016 and 2017)

Urban guppies had more offspring than non‐urban guppies (13 ± 0.5 and 8 ± 0.5, respectively; GLMM, *z* = 2.1, *p* = 0.04; Figure 3a; Tables S5 and S6). Although Figure 3a suggests an effect of biodiversity on fecundity, the GLMM model did not show a significant interaction between the two. This is likely an effect of body size which was strongly and positively correlated with fecundity (GLMM, *z* = 49.8, *p* < 0.001), while guppy density is related to a small reduction in fecundity (GLMM, *z* = −6, *p* < 0.001). Although there was a tendency for increased gonad mass and reproductive allotment in urban guppies, there were no statistically significant differences between urban and non‐urban guppies, or between reaches that differed in fish diversity (Figure S3). Urban guppies had better condition compared to non‐urban guppies regardless of biodiversity (3.4 ± 0.1 and 2 ± 0.1 *I* index, respectively; Figure 3b). Overall guppy condition was slightly better in 2016 compared to 2017 (3.3 ± 0.1 and 2.6 ± 0.1, respectively; GLMM, *t* = −9.4, *p* < 0.001). Reaches with denser guppy populations also had guppies with higher condition, but the effect was small (GLMM, *t* = 4.4, *p* < 0.01; Tables S5 and S6).

### 3.3 Mechanism of change to guppy life‐history traits and the effect of biodiversity

**Figure 4:**
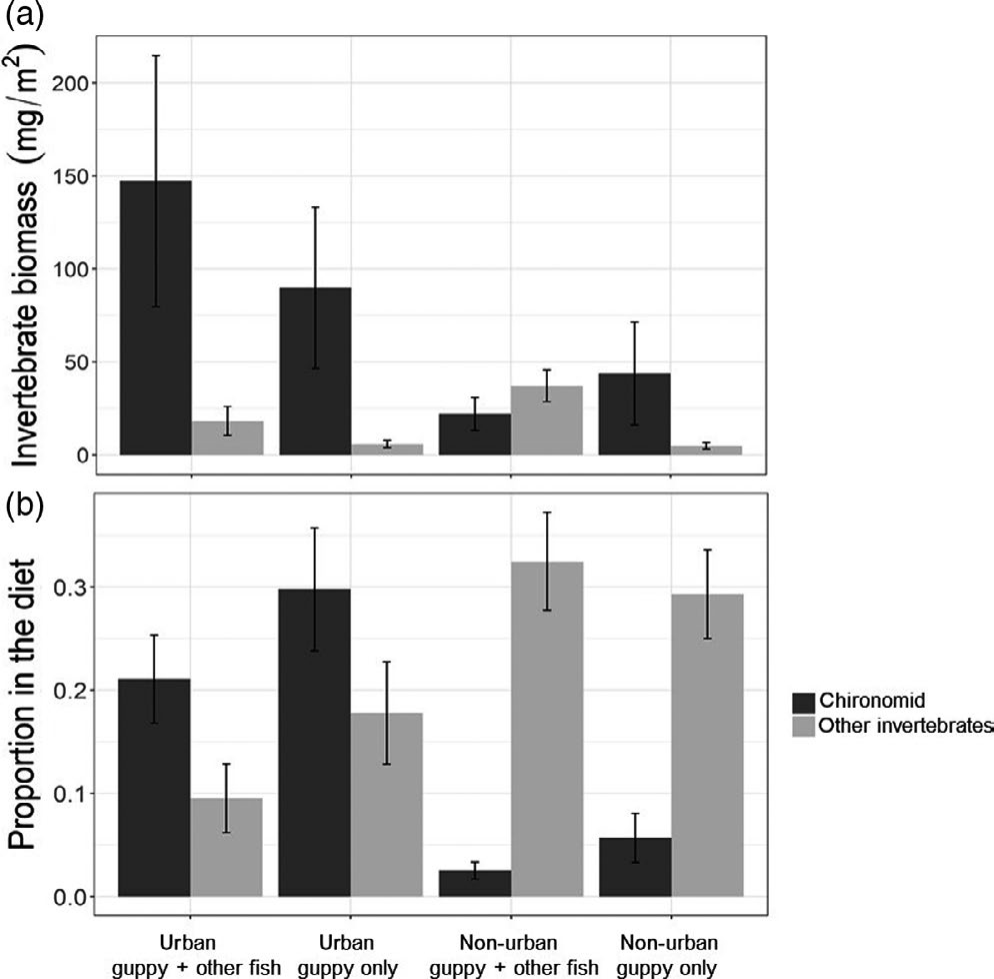
Chironomids (dark grey) and other invertebrates (light grey) that were measured in the streams as biomass available for consumption (panel a) and found in the guts of female guppies, expressed as proportion of total diet (panel b). The data include samples of urban reaches where guppies occur with other fish species (urban:guppy + other fish), urban reaches where guppies are the only fish species (urban:guppy only, non‐urban reaches where guppies occur with other fish species (non‐urban:guppy + other fish) and non‐urban reaches where guppies are the only fish species (non‐urban:guppy only). The category of other invertebrates includes insect families within Diptera, Lepidoptera and Trichoptera and Oligochaeta. Bars represent the mean and lines are the standard error of the mean. Data shown include both sampling years 2016 and 2017

Urbanization increased the availability of food for guppies. The estimated biomass of all invertebrate taxa together was higher in urban reaches compared to non‐urban reaches, regardless of fish biodiversity (122 ± 40 and 38 ± 9 mg/m^2^, respectively; GLMM, *t* = 2.9, *p* < 0.01; Figure 4a; Table S4). The estimated biomass of chironomids was also higher in urban reaches than in non‐urban reaches, regardless of fish biodiversity (114 ± 40 and 19 ± 5 mg/m^2^, respectively; GLMM, *t* = 3.7, *p* < 0.001; Figure 4a; Table S4). There were no significant differences in the estimated biomass of other invertebrates between urban and non‐urban reaches, independent of fish diversity (Figure 4a).

Guppy diets were dominated by detritus regardless of urbanization or fish diversity (proportion = 0.5–0.7 of gut contents). Invertebrates were the second most important diet items, with proportion of 0.3–0.5 of the gut contents, with no clear differences between urban or non‐urban reaches, or with differences in fish diversity (Figure S4). However, chironomids were consumed in higher proportion in urban reaches than in non‐urban reaches, regardless of fish biodiversity (0.25 ± 0.04 and 0.04 ± 0.01, respectively; GLMM, *z* = 3.8, *p* < 0.001). The consumption of chironomids was slightly reduced at higher guppy density (GLMM, *z* = −2.8, *p* = 0.01). The consumption of other invertebrates was lower in urban than non‐urban reaches (GLMM, *z* = −1.92, *p* = 0.05) specially in 2017 (GLMM, *z* = −2.59, *p* < 0.01) independent of fish biodiversity (Figure 4b; Table S6).

**Figure 5:**
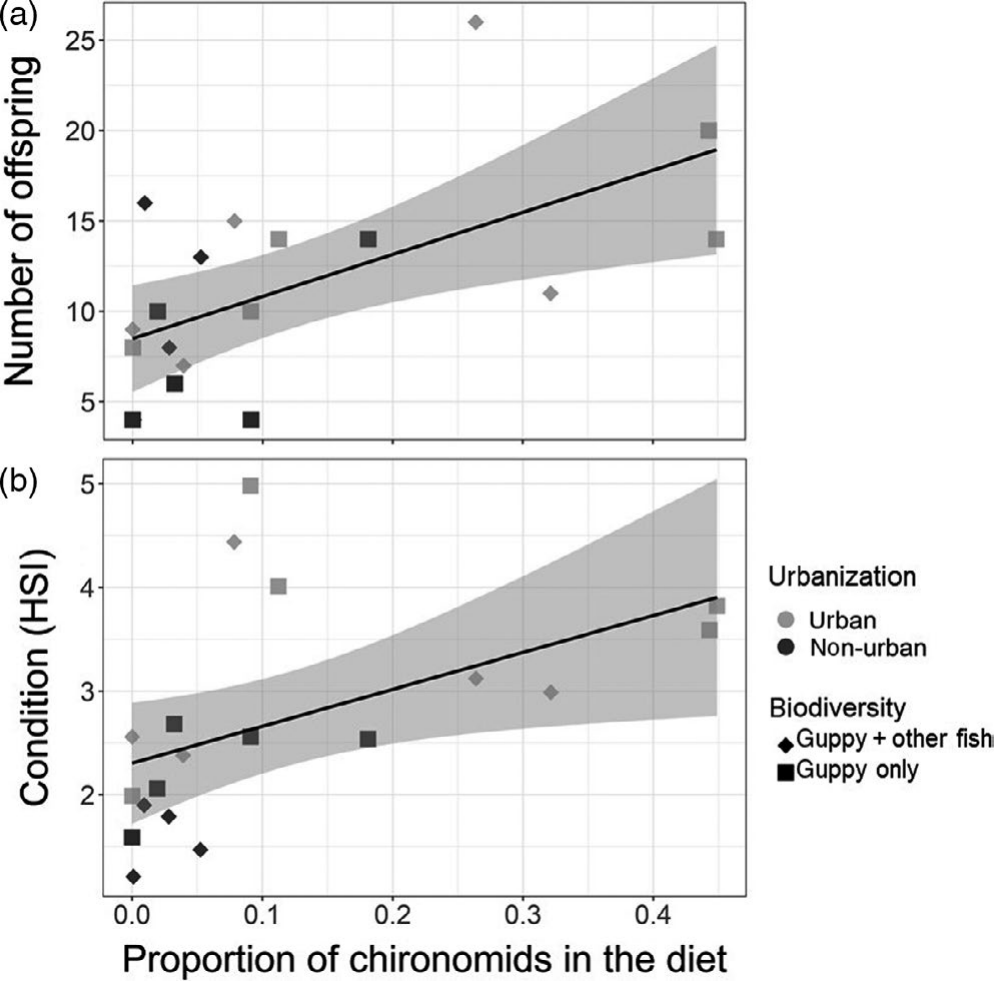
Relationship between diet (proportion of chironomids consumed) and (a) number of offspring, (b) condition. Each symbol represents the average value in urban (grey) and non‐urban (black) reaches where guppies co‐occur with competitors and predators (high biotic interactions, diamonds) and reaches where guppies are the only fish species (low biotic interactions, squares). Lines represent the linear model fit and shades are confidence intervals. Model results are in the Supporting Information (Table S8)

A relationship was observed between diet versus guppy fecundity and diet versus guppy condition. Following model selection, the best LMM models ($R_{\text{adj}}^{2}$ = 77–82) suggested that the consumption of chironomids had a positive relationship with fecundity (coef_avg_ = 19.48). This model also showed that fecundity increased with body size (coef_avg_ = 1.58) and declined when other fish species were present (coef_avg_ = −0.66; Figure 5a). Fecundity was also elevated in 2017, compared to 2016 (coef_avg_ = 1.18; Table S7). The best models ($R_{\text{adj}}^{2}$ = 26%–36%) also suggested that the consumption of chironomids (coef_avg_ = 3.35) had an overall positive effect on guppy condition, despite an overall reduction in condition in 2017 (coef_avg_ = −0.19; Figure 5b; Table S7). The likelihood ratio test showed that the proportion of chironomid consumed was a significant predictor of fecundity (*χ* ^2^ = 17.8, *df* = 1, *p* < 0.001) and condition (*χ* ^2^ = 3.8, *df* = 1, *p* = 0.05). We could not confidently assess the relationship between diet and body length because, following model selection, the null model was included as one of the top models. A table with the full model selection for each LMM model can be found in the supplement (Table S8).

## 4 DISCUSSION

Invasive species are pervasive in urban systems, but the mechanisms that facilitate invasion and success are not clear (Cadotte et al., 2017). Here we show evidence that guppies, a global invasive species, proliferate in urban ecosystems, reaching high densities, and that urbanization enhances traits that are likely to make guppies good invaders. Our data suggest that the relaxation of life‐history trade‐offs can be an important mechanism facilitating the success of invasive species in urban systems. Differences between urban and non‐urban guppies vary slightly between years and are slightly dampened by the presence of other fish species.

Urbanization causes profound environmental changes to ecosystems. The streams of Rio de Janeiro show some of the symptoms of the ‘urban stream syndrome’ (Walsh et al., 2005), including high concentration of ammonium. In addition, these streams are especially contaminated with faecal coliforms, exceeding by 196 fold the maximum concentration allowed by local regulations (Conselho Nacional de Meio Ambiente ‐ CONAMA, 2000). This high coliform concentration likely results from the discharge of raw sewage because of the poor sewer infrastructure (Borges, Santos, Caldas, & Lapa, 2015).

Despite the degraded environment, invaders can be more successful in urban than in non‐urban systems. Guppies in urban streams have higher population density and body sizes than guppies in non‐urban streams (Figure 2). Urban invaders often maintain higher population density in urban than in nearby non‐urban systems (Shochat et al., 2010). However, whether urban invaders also maintain increased body sizes in urban than in non‐urban populations is debated. For instance, house sparrows *Passer domesticus*, have reduced body sizes in urban systems (Meillère, Brischoux, Parenteau, & Angelier, 2015). Conversely, rats *Rattus norvegicus* and *Rattus rattus*, can have larger body sizes in urban than in non‐urban systems (Feng & Himsworth, 2014). Larger body size can help invaders to establish populations (Schröder et al., 2009). In guppies, larger body size as a response to predation release, has been shown to help individuals resist high waterflow and flash floods (Hockley, Wilson, Brew, & Cable, 2014). Thus, larger body size can improve population's persistence in flashy urban streams. Larger body size also can help guppies and other fish to travel longer distances facilitating dispersal (Brown, 1985; Croft et al., 2003). Increased body size associated with better condition allow females to have more offspring which can promote the establishment of new populations far from the site of first introduction (Liu, Comte, & Olden, 2017).

Our data suggest that urbanization can facilitate the success of invaders by increasing food availability. Urban streams have increased chironomid biomass (Figure 4). If compared to guppy's native non‐urban streams in the Southern range of Trinidad, our urban reaches have more than 10 times the biomass of chironomids (10 ± 1 mg/m^2^ and 114 ± 40, respectively; Eugenia Zandonà personal communication). Chironomids are a principal food for guppies (Ganassin et al., 2019; Zandonà et al., 2011). Therefore, one of the ways in which urbanization facilitates guppy success is by increasing the biomass and consumption of chironomids which are highly nutritious. Such mechanism is likely to be common because chironomids are often found in large densities in aquatic urban systems worldwide and guppies, as well as other urban dwellers, have been shown to take advantage of this food resource (Kelly, Cuevas, & Ramírez, 2019; Moreyra & Padovesi‐Fonseca, 2015; Naidoo, Vosloo, & Schoeman, 2013; Sterling, Rosemond, & Wenger, 2016). Urbanization is expected to increase food availability in both aquatic and terrestrial systems (El‐Sabaawi, 2018). Thus, it is likely that different invasive species can take advantage of the increased food availability in urban systems to enhance their success.

Urbanization potentially relaxes life‐history trade‐offs of invasive species. Nutrition shapes life‐history traits across taxa through trade‐offs between nutritional investment in somatic growth, reproduction and energy required for biotic interactions (Stearns, 1989; Swanson et al., 2016). For example, studies on non‐urban streams in Trinidad show that guppies have smaller body size and less offspring when population density is high (a result of predation release), likely due to competition for limited food resources (Bassar et al., 2016). However, increased food resources in human altered systems have been hypothesized to relax life‐history trade‐offs (Snell‐Rood et al., 2015). Our data provide preliminary support for this hypothesis by showing that urban guppies have increased body sizes, more offspring and better condition than non‐urban guppies even when density is high, independent of fish biodiversity (Figures 2 and 3). However, to clearly demonstrate the relaxation of trade‐offs requires estimation of mortality and growth rates because they further help explain the patterns of resource allocation (Roff, Heibo, & Vøllestad, 2006). Further studies under controlled condition can help understand the causal relationship between food availability, diet and the life history of urban invaders.

The value of conserving biodiversity in cities is debated because the ecological and human benefits are not always clear (Dearborn & Kark, 2010). A potential benefit of urban biodiversity is to provide biotic resistance against invasion, as seen in non‐urban systems (Kennedy et al., 2002). However, such protective role of biodiversity is not clear from our data. The presence of other fish has a small, non‐significant effect on guppy population density, number of offspring and condition, although these effects are always in a negative direction, suggesting protection from invasion. We assessed the effects of biodiversity using categories, but other aspects of biodiversity, such as species identity, dominance and rarity can also be important to determine its protective role against invasion in urban streams (Henriksson, Yu, Wardle, & Englund, 2015).

An important question for future studies is whether adaptive traits that increase invasiveness are plastic (non‐heritable) or evolutionary (heritable). Urbanization is increasingly shown to be a strong selective agent for trait change which can cause contemporary evolution across many taxa (Alberti, 2015). Studies on guppies in non‐urban streams in their native range suggest that body size and the number of offspring have a heritable component, but there is also considerable plasticity, especially in body size (Gordon, Hendry, & Reznick, 2017; Reznick & Bryga, 1987). In the future, laboratory breeding experiments could test if urbanization drives the evolution of increased body sizes and ‘fast’ life‐history traits in guppies and other urban invaders. This information can be fundamental for managers because the evolution of a highly invasive phenotype can promote range expansion and secondary invasions in urban and non‐urban systems (Bertelsmeier & Keller, 2018).

Globally distributed invasive species that flourish in urban environments such as guppies, offer a great opportunity to study how predictable is the adaptation to urban environments (Donihue & Lambert, 2015). Urbanization is expected to have similar effects on ecosystems worldwide (McKinney, 2006). Thus, it is likely that urban invaders can be used in a globally replicated experiment to test the repeatability and pace of adaptation (Johnson & Munshi‐South, 2017). The results of our study for example, can possibly be used to test whether the mechanism of adaptation seen in guppies is repeatable among cities and different species across the world.

## AUTHORS' CONTRIBUTIONS

P.S.M., E.Z., R.M. and R.E.‐S. conceived the ideas and designed methodology; P.S.M., L.R.M. and T.C.F. collected the data; P.S.M., T.C.F. and R.E.‐S. analysed the data; P.S.M. and R.E.‐S. led the writing of the manuscript. All authors contributed critically to the drafts and gave final approval for publication.

## Supporting information

Supplementary Material

## Data Availability

Data are available from Scholars Portal Dataverse: https://doi.org/10.5683/SP2/XTK0LQ (Marques, Manna, Frauendorf, Zandonà, & El‐Sabaawi, 2020).

## References

[jane13293-bib-0001] Alberti, M. (2015). Eco‐evolutionary dynamics in an urbanizing planet. Trends in Ecology & Evolution, 30(2), 114–126. 10.1016/j.tree.2014.11.007 25498964

[jane13293-bib-0002] Alberti, M. , Correa, C. , Marzluff, J. M. , Hendry, A. P. , Palkovacs, E. P. , Gotanda, K. M. , … Zhou, Y. (2017). Global urban signatures of phenotypic change in animal and plant populations. Proceedings of the National Academy of Sciences of the United States of America, 114(34), 8951–8956. 10.1073/pnas.1606034114 28049817 PMC5576774

[jane13293-bib-0003] Allen, W. L. , Street, S. E. , & Capellini, I. (2017). Fast life history traits promote invasion success in amphibians and reptiles. Ecology Letters, 20(2), 222–230. 10.1111/ele.12728 28052550 PMC6849728

[jane13293-bib-0004] Aronson, M. F. J. , Handel, S. N. , & Clemants, S. E. (2007). Fruit type, life form and origin determine the success of woody plant invaders in an urban landscape. Biological Invasions, 9(4), 465–475. 10.1007/s10530-006-9053-1

[jane13293-bib-0005] Bartoń, K. (2018). Package MuMIn: multi‐model inference. R Package version 1.42.1. https://cran.r‐project.org/web/packages/MuMIn/MuMIn.pdf

[jane13293-bib-0006] Bassar, R. D. , Childs, D. Z. , Rees, M. , Tuljapurkar, S. , Reznick, D. N. , & Coulson, T. (2016). The effects of asymmetric competition on the life history of Trinidadian guppies. Ecology Letters, 19(3), 268–278. 10.1111/ele.12563 26843397 PMC4991285

[jane13293-bib-0007] Bastos, R. F. , Condini, M. V. , Varela Junior, A. S. , & Garcia, A. M. (2011). Diet and food consumption of the pearl cichlid *Geophagus brasiliensis* (Teleostei: Cichlidae): Relationships with gender and sexual maturity. Neotropical Ichthyology, 9(4), 825–830. 10.1590/S1679-62252011005000049

[jane13293-bib-0008] Bates, D. M. , Machler, M. , Bolker, B. M. , & Walker, S. C. (2015). Fitting linear mixed‐effects models using lme4. Journal of Statistical Software, 67(1). 10.18637/jss.v067.i01

[jane13293-bib-0009] Benke, A. C. , Huryn, A. D. , Smock, L. A. , & Wallace, J. B. (1999). Length–mass relationships for freshwater macroinvertebrates in North America with particular reference to the Southeastern United States. Journal of the North American Benthological Society, 18(3), 308–343. 10.2307/1468447

[jane13293-bib-0010] Bernhardt, E. S. , Band, L. E. , Walsh, C. J. , & Berke, P. E. (2008). Understanding, managing, and minimizing urban impacts on surface water nitrogen loading. Annals of the New York Academy of Sciences, 1134(1), 61–96. 10.1196/annals.1439.014 18566090

[jane13293-bib-0011] Bertelsmeier, C. , & Keller, L. (2018). Bridgehead effects and role of adaptive evolution in invasive populations. Trends in Ecology & Evolution, 33(7), 527–534. 10.1016/j.tree.2018.04.014 29764688

[jane13293-bib-0012] Bonato, K. O. , Delariva, R. L. , & da Silva, J. C. (2012). Diet and trophic guilds of fish assemblages in two streams with different anthropic impacts in the northwest of Paraná, Brazil. Zoologia (Curitiba), 29(1), 27–38. 10.1590/S1984-46702012000100004

[jane13293-bib-0013] Borges, R. C. , Santos, F. V. , Caldas, V. G. , & Lapa, C. M. F. (2015). Use of geographic information system (GIS) in the characterization of the Cunha Canal, Rio de Janeiro, Brazil: Effects of the urbanization on water quality. Environmental Earth Sciences, 73(3), 1345–1356. 10.1007/s12665-014-3493-1

[jane13293-bib-0014] Brown, K. L. (1985). Demographic and genetic characteristics of dispersal in the mosquitofish, *Gambusia affinis* (Pisces: Poeciliidae). Copeia, 5, 597–612. 10.2307/1444750

[jane13293-bib-0015] Burnham, K. P. , & Anderson, D. R. (2002). Model selection and multimodel inference: A practical information‐theoretic approach (2nd ed.). New York, NY: Springer‐Verlag.

[jane13293-bib-0016] Byers, J. E. (2002). Impact of non‐indigenous species on natives enhanced by anthropogenic alteration of selection regimes. Oikos, 97(3), 449–458. 10.1034/j.1600-0706.2002.970316.x

[jane13293-bib-0017] Cadotte, M. W. , Yasui, S. L. E. , Livingstone, S. , & MacIvor, J. S. (2017). Are urban systems beneficial, detrimental, or indifferent for biological invasion? Biological Invasions, 19(12), 3489–3503. 10.1007/s10530-017-1586-y

[jane13293-bib-0018] Capellini, I. , Baker, J. , Allen, W. L. , Street, S. E. , & Venditti, C. (2015). The role of life history traits in mammalian invasion success. Ecology Letters, 18(10), 1099–1107. 10.1111/ele.12493 26293900 PMC4989474

[jane13293-bib-0019] Carle, F. L. , & Strub, M. R. (1978). A new method for estimating population size from removal. Biometrics, 34(4), 621–630.

[jane13293-bib-0020] Carrete, M. , & Tella, J. L. (2011). Inter‐individual variability in fear of humans and relative brain size of the species are related to contemporary urban invasion in birds. PLoS ONE, 6(4), e18859 10.1371/journal.pone.0018859 21526193 PMC3079730

[jane13293-bib-0021] Conselho Nacional de Meio Ambiente ‐ CONAMA . (2000). Resolução 274 de 29 de novembro de 2000. Brasília, Brazil: Estabelece condições de balneabilidade das águas brasileiras.

[jane13293-bib-0022] Croft, D. P. , Albanese, B. , Arrowsmith, B. J. , Botham, M. , Webster, M. , & Krause, J. (2003). Sex‐biased movement in the guppy (*Poecilia reticulata*). Oecologia, 137(1), 62–68. 10.1007/s00442-003-1268-6 12856201

[jane13293-bib-0023] Dagar, A. , Zilberg, D. , Cohen, Z. , Boussiba, S. , & Khozin‐Goldberg, I. (2010). Short‐term dietary supplementation with the microalga *Parietochloris incisa* enhances stress resistance in guppies *Poecilia reticulata* . Aquaculture Research, 41(2), 267–277. 10.1111/j.1365-2109.2009.02329.x

[jane13293-bib-0024] Dearborn, D. C. , & Kark, S. (2010). Motivations for conserving urban biodiversity. Conservation Biology, 24(2), 432–440. 10.1111/j.1523-1739.2009.01328.x 19775276

[jane13293-bib-0025] Deus, C. P. , & Petrere‐Junior, M. (2003). Seasonal diet shifts of seven fish species in an Atlantic rainforest stream in Southeastern Brazil. Brazilian Journal of Biology, 63(4), 579–588. 10.1590/S1519-69842003000400005 15029369

[jane13293-bib-0026] Donihue, C. M. , & Lambert, M. R. (2015). Adaptive evolution in urban ecosystems. Ambio, 44(3), 194–203. 10.1007/s13280-014-0547-2 25056615 PMC4357625

[jane13293-bib-0027] Edberg, S. C. , Rice, E. W. , Karlin, R. J. , & Allen, M. J. (2000). *Escherichia coli*: The best biological drinking water indicator for public health protection. Journal of Applied Microbiology, 88(S1), 106S–116S. 10.1111/j.1365-2672.2000.tb05338.x 10880185

[jane13293-bib-0028] El‐Sabaawi, R. (2018). Trophic structure in a rapidly urbanizing planet. Functional Ecology, 32(7), 1718–1728. 10.1111/1365-2435.13114

[jane13293-bib-0029] El‐Sabaawi, R. W. , Marshall, M. C. , Bassar, R. D. , López‐Sepulcre, A. , Palkovacs, E. P. , & Dalton, C. (2015). Assessing the effects of guppy life history evolution on nutrient recycling: From experiments to the field. Freshwater Biology, 60(3), 590–601. 10.1111/fwb.12507

[jane13293-bib-0030] Faeth, S. H. , Bang, C. , & Saari, S. (2011). Urban biodiversity: Patterns and mechanisms. Annals of the New York Academy of Sciences, 1223(1), 69–81. 10.1111/j.1749-6632.2010.05925.x 21449966

[jane13293-bib-0031] Feng, A. Y. T. , & Himsworth, C. G. (2014). The secret life of the city rat: A review of the ecology of urban Norway and black rats (*Rattus norvegicus* and *Rattus rattus*). Urban Ecosystems, 17(1), 149–162. 10.1007/s11252-013-0305-4

[jane13293-bib-0032] Fox, J. , & Weisberg, S. (2011). A R companion to applied regression (2nd ed.). Thousand Oaks, CA: Sage.

[jane13293-bib-0033] Ganassin, M. J. M. , Frota, A. , Muniz, C. M. , Baumgartner, M. T. , & Hahn, N. S. (2019). Urbanisation affects the diet and feeding selectivity of the invasive guppy *Poecilia reticulata* . Ecology of Freshwater Fish, 1–14. 10.1111/eff.12511

[jane13293-bib-0034] Gordon, S. P. , Hendry, A. P. , & Reznick, D. N. (2017). Predator‐induced contemporary evolution, phenotypic plasticity, and the evolution of reaction norms in Guppies. Copeia, 105(3), 514–522. 10.1643/CE-16-522

[jane13293-bib-0035] Grueber, C. E. , Nakagawa, S. , Laws, R. J. , & Jamieson, I. G. (2011). Multimodel inference in ecology and evolution: Challenges and solutions. Journal of Evolutionary Biology, 24(4), 699–711. 10.1111/j.1420-9101.2010.02210.x 21272107

[jane13293-bib-0036] Haag‐Wackernagell, D. , & Moch, H. (2004). Health hazards posed by feral pigeons. Journal of Infection, 48(4), 307–313. 10.1016/j.jinf.2003.11.001 15066331

[jane13293-bib-0037] Haynes, J. L. (1995). Standardized classification of poeciliid development for life‐history studies. Copeia, 1, 147–154. 10.2307/1446809

[jane13293-bib-0038] Henriksson, A. , Yu, J. , Wardle, D. A. , & Englund, G. (2015). Biotic resistance in freshwater fish communities: Species richness, saturation or species identity? Oikos, 124(8), 1058–1064. 10.1111/oik.01700

[jane13293-bib-0039] Hockley, F. A. , Wilson, C. A. M. , Brew, A. , & Cable, J. (2014). Fish responses to flow velocity and turbulence in relation to size, sex and parasite load. Journal of the Royal Society Interface, 11(91). 10.1098/rsif.2013.0814 PMC3869156 24284893

[jane13293-bib-0040] Holmes, R. M. , Aminot, A. , Kérouel, R. , Hooker, B. A. , & Peterson, B. J. (1999). A simple and precise method for measuring ammonium in marine and freshwater ecosystems. Canadian Journal of Fisheries and Aquatic Sciences, 56(10), 1801–1808. 10.1139/f99-128

[jane13293-bib-0041] Hothorn, T. , Zeileis, A. , Farebrother, R. W. , Cummins, C. , Millo, G. , & Mitchell, D. (2018). Testing linear regression models (version 0.9‐36). Retrieved from https://cran.r‐project.org/web/packages/lmtest/lmtest.pdf

[jane13293-bib-0042] Johnson, M. T. J. , & Munshi‐South, J. (2017). Evolution of life in urban environments. Science, 358(6363), eaam8327. 10.1126/science.aam8327 29097520

[jane13293-bib-0043] Kassambara, A. , & Mundt, F. (2016). factoextra: Extract and visualize the results of multivariate data analyses (version 1.0.5). Retrieved form https://cran.r‐project.org/web/packages/factoextra/factoextra.pdf

[jane13293-bib-0044] Kelly, S. P. , Cuevas, E. , & Ramírez, A. (2019). Urbanization increases the proportion of aquatic insects in the diets of riparian spiders. Freshwater Science, 38(2), 379–390. 10.1086/703442

[jane13293-bib-0045] Kennedy, T. A. , Naeem, S. , Howe, K. M. , Knops, J. M. H. , Tilman, D. , & Reich, P. (2002). Biodiversity as a barrier to ecological invasion. Nature, 417(6889), 636–638. 10.1038/nature00776 12050662

[jane13293-bib-0046] Levine, J. M. , & D'Antonio, C. M. (1999). Elton revisited: A review of evidence linking diversity and invasibility. Oikos, 87(1), 15 10.2307/3546992

[jane13293-bib-0047] Liu, C. , Comte, L. , & Olden, J. D. (2017). Heads you win, tails you lose: Life‐history traits predict invasion and extinction risk of the world's freshwater fishes. Aquatic Conservation: Marine and Freshwater Ecosystems, 27(4), 773–779. 10.1002/aqc.2740

[jane13293-bib-0048] Lloret, J. , Shulman, G. , & Love, M. R. (2014). Condition and health indicators of exploited marine fishes. Oxford, UK: Wiley Blackwell.

[jane13293-bib-0049] Lososová, Z. , Chytrý, M. , Danihelka, J. , Tichý, L. , & Ricotta, C. (2016). Biotic homogenization of urban floras by alien species: The role of species turnover and richness differences. Journal of Vegetation Science, 27(3), 452–459. 10.1111/jvs.12381

[jane13293-bib-0050] Mackintosh, T. J. , Davis, J. A. , & Thompson, R. M. (2017). The effects of urbanization on trophic relationships in constructed wetlands. Freshwater Science, 36(1), 138–150. 10.1086/690674

[jane13293-bib-0051] Magurran, A. E. (2005). Evolutionary ecology: The Trinidadian guppy. Oxford, UK: Oxford University Press.

[jane13293-bib-0087] Marques, P. , Manna, L. R. , Frauendorf, T. C. , Zandonà, E. , & El‐Sabaawi, R. (2020). Replication data for "Urbanization can increase the invasive potential of alien species". Scholars Portal Dataverse. 10.5683/SP2/XTK0LQ PMC7590067 32627190

[jane13293-bib-0052] McKinney, M. L. (2006). Urbanization as a major cause of biotic homogenization. Biological Conservation, 127(3), 247–260. 10.1016/j.biocon.2005.09.005

[jane13293-bib-0053] Meillère, A. , Brischoux, F. , Parenteau, C. , & Angelier, F. (2015). Influence of urbanization on body size, condition, and physiology in an urban exploiter: A multi‐component approach. PLoS ONE, 10(8), e0135685 10.1371/journal.pone.0135685 26270531 PMC4535910

[jane13293-bib-0054] Merritt, R. W. , & Cummins, K. W. (1996). An introduction to the aquatic insects of North America. Dubuque, IA: Kendall Hunt.

[jane13293-bib-0055] Moreyra, A. K. , & Padovesi‐Fonseca, C. (2015). Environmental effects and urban impacts on aquatic macroinvertebrates in a stream of central Brazilian Cerrado. Sustainable Water Resources Management, 1(2), 125–136. 10.1007/s40899-015-0013-8

[jane13293-bib-0056] Mugnai, R. , Nessimian, J. L. , & Baptista, D. F. (2010). Manual de identificação de macroinvertebrados aquáticos do Estado do Rio de Janeiro. Rio de Janeiro: Technical Books.

[jane13293-bib-0057] Naidoo, S. , Vosloo, D. , & Schoeman, M. C. (2013). Foraging at wastewater treatment works increases the potential for metal accumulation in an urban adapter, the banana bat (*Neoromicia nana*). African Zoology, 48(1), 39–55. 10.1080/15627020.2013.11407567

[jane13293-bib-0058] Neumann, R. M. , & Allen, M. S. (2007). Size structure In Guy C. S. & Brown M. L. (Eds.), Analysis and interpretation of freshwater fisheries data (p. 961). Bethesda, MD: American Fisheries Society.

[jane13293-bib-0059] Ogle, D. H. (2015). Introductory fisheries analyses with R (1st ed.). Boca Raton, FL: Chapman and Hall/CRC.

[jane13293-bib-0060] Ogle, D. , Wheeler, P. , & Dino, A. (2018). FSA: Fisheries stock analysis (version 0.8.22.9000) [R package]. Retrieved from https://github.com/droglenc/FSA

[jane13293-bib-0061] R Core Team . (2018). R: A language and environment for statistical computing (version 3.5.1). Vienna, Austria: R Foundation for Statistical Computing.

[jane13293-bib-0062] Reznick, D. N. , & Bryga, H. (1987). Life‐history evolution in guppies (*Poecilia reticulata*): 1. Phenotypic and genetic changes in an introduction experiment. Evolution, 41(6), 1370 10.2307/2409101 28563598

[jane13293-bib-0063] Reznick, D. , Butler IV, M. J. , & Rodd, H. (2001). Life‐history evolution in guppies. VII. The comparative ecology of high‐ and low‐predation environments. The American Naturalist, 157(2), 126–140. 10.1086/318627 18707267

[jane13293-bib-0064] Reznick, D. , & Endler, J. A. (1982). The impact of predation on life history evolution in Trinidadian guppies (*Poecilia reticulata*). Evolution, 36(1), 160 10.2307/2407978 28581096

[jane13293-bib-0065] Rice, E. W. , Baird, R. B. , Eaton, A. D. , & Clesceri, L. S. (Eds.) (2012). Standard methods for the examination of water and wastewater (22nd ed.). Washington, DC: American Public Health Association, American Water Works Association, Water Environment Federation.

[jane13293-bib-0066] Richardson, D. M. , Pysek, P. , & Carlton, J. T. (2011). A compendium of essential concepts and terminology in invasion ecology In Fifty years of invasion ecology: The legacy of Charles Elton (p. 409). Oxford, UK: Wiley‐Blackwell 10.1002/9781444329988

[jane13293-bib-0068] Rocha, C. F. D. , Bergallo, H. G. , & Mazzoni, R. (2011). Invasive vertebrates in Brazil In Pimentel D. (Ed.), Biological invasions: Economic and environmental costs of alien plant, animal, and microbe species (2nd ed., pp. 53–103). Boca Raton, FL: CRC Press.

[jane13293-bib-0069] Roff, D. A. (1992). The evolution of life histories: Theory and analysis. New York, NY: Chapman and Hall.

[jane13293-bib-0070] Roff, D. A. , Heibo, E. , & Vøllestad, L. A. (2006). The importance of growth and mortality costs in the evolution of the optimal life history. Journal of Evolutionary Biology, 19(6), 1920–1930. 10.1111/j.1420-9101.2006.01155.x 17040389

[jane13293-bib-0071] Schröder, A. , Nilsson, K. A. , Persson, L. , Kooten, T. V. , & Reichstein, B. (2009). Invasion success depends on invader body size in a size‐structured mixed predation–competition community. Journal of Animal Ecology, 78(6), 1152–1162. 10.1111/j.1365-2656.2009.01590.x 19682142

[jane13293-bib-0072] Sekhon, J. S. (2011). Multivariate and propensity score matching with balance optimization: The matching package for R. Journal of Statistical Software, 42(7), 1–52.

[jane13293-bib-0073] Shik, J. Z. , & Dussutour, A. (2020). Nutritional dimensions of invasive success. Trends in Ecology & Evolution. 10.1016/j.tree.2020.03.009 32668214

[jane13293-bib-0074] Shochat, E. , Lerman, S. B. , Anderies, J. M. , Warren, P. S. , Faeth, S. H. , & Nilon, C. H. (2010). Invasion, competition, and biodiversity loss in urban ecosystems. BioScience, 60(3), 199–208. 10.1525/bio.2010.60.3.6

[jane13293-bib-0075] Snell‐Rood, E. , Cothran, R. , Espeset, A. , Jeyasingh, P. , Hobbie, S. , & Morehouse, N. I. (2015). Life‐history evolution in the anthropocene: Effects of increasing nutrients on traits and trade‐offs. Evolutionary Applications, 8(7), 635–649. 10.1111/eva.12272 26240602 PMC4516417

[jane13293-bib-0076] Stearns, S. C. (1989). Trade‐offs in life‐history evolution. Functional Ecology, 3(3), 259–268. 10.2307/2389364

[jane13293-bib-0077] Stearns, S. C. (1992). The evolution of life histories (1st ed.). Oxford, UK; New York, NY: Oxford University Press.

[jane13293-bib-0078] Sterling, J. L. , Rosemond, A. D. , & Wenger, S. J. (2016). Watershed urbanization affects macroinvertebrate community structure and reduces biomass through similar pathways in Piedmont streams, Georgia, USA. Freshwater Science, 35(2), 676–688. 10.1086/686614

[jane13293-bib-0079] Swanson, E. M. , Espeset, A. , Mikati, I. , Bolduc, I. , Kulhanek, R. , White, W. A. , … Snell‐Rood, E. C. (2016). Nutrition shapes life‐history evolution across species. Proceedings of the Royal Society B: Biological Sciences, 283(1834). 10.1098/rspb.2015.2764 PMC4947880 27412282

[jane13293-bib-0080] Travis, J. , Reznick, D. , Bassar, R. D. , López‐Sepulcre, A. , Ferriere, R. , & Coulson, T. (2014). Do eco‐evo feedbacks help us understand nature? Answers from studies of the Trinidadian guppy. Advances in Ecological Research, 50, 1–40. 10.1016/B978-0-12-801374-8.00001-3

[jane13293-bib-0081] Violle, C. , Navas, M.‐L. , Vile, D. , Kazakou, E. , Fortunel, C. , Hummel, I. , & Garnier, E. (2007). Let the concept of trait be functional! Oikos, 116(5), 882–892. 10.1111/j.0030-1299.2007.15559.x

[jane13293-bib-0082] Walsh, C. J. , Roy, A. H. , Feminella, J. W. , Cottingham, P. D. , Groffman, P. M. , & Morgan, R. P. (2005). The urban stream syndrome: Current knowledge and the search for a cure. Journal of the North American Benthological Society, 24(3), 706–723. 10.1899/04-028.1

[jane13293-bib-0083] Wickham, H. (2009). ggplot2: Elegant graphics for data analysis (2nd ed.). New York, NY: Springer‐Verlag.

[jane13293-bib-0084] Wilson, J. R. U. , Dormontt, E. E. , Prentis, P. J. , Lowe, A. J. , & Richardson, D. M. (2009). Something in the way you move: Dispersal pathways affect invasion success. Trends in Ecology & Evolution, 24(3), 136–144. 10.1016/j.tree.2008.10.007 19178981

[jane13293-bib-0085] Yule, C. M. , Gan, J. Y. , Jinggut, T. , & Lee, K. V. (2015). Urbanization affects food webs and leaf‐litter decomposition in a tropical stream in Malaysia. Freshwater Science, 34(2), 702–715. 10.1086/681252

[jane13293-bib-0086] Zandonà, E. , Auer, S. K. , Kilham, S. S. , Howard, J. L. , López‐Sepulcre, A. , O'Connor, M. P. , … Reznick, D. N. (2011). Diet quality and prey selectivity correlate with life histories and predation regime in Trinidadian guppies. Functional Ecology, 25(5), 964–973. 10.1111/j.1365-2435.2011.01865.x

