# Supplementary material for: Urbanization can increase the invasive potential of alien species
Source: J Anim Ecol. 2020 Jul 28;89(10):2345–55. doi: 10.1111/1365-2656.13293 (PMC7590067; doi:10.1111/1365-2656.13293)
Supplement: Supplementary file 1 — Supplementary Material [file JANE-89-2345-s001.docx]

**Supporting information**

Table S1. List of fish species that co-occur with guppies in urban and non-urban reaches in the sites AntesComu (ANC), Floresta (FLOR), Water planet sujo (WPS), Camorim limpo (CAML), Tingui (TNG), Ubatiba (UBA) in Rio de Janeiro, Brazil. Where, Asty=*Astyanax* sp., Call=*Callichthys callichthys*, Char=Characidium sp., Geop=*Geophagus brasiliensis*, Gymn=*Gymnotus* sp., Hopl=*Hoplias malabaricus*, Hypo=*Hypostomus* sp., Mima=*Mimagoniates microlepis*, Paro=*Parotocinclus* sp., Phal=*Palloceros* sp., Pime=*Pimelodella lateristriga*, Rham=*Rhamdia quelen*, Scle=*Scleromystax* sp., Synb=*Synbranchus* sp., Tric=*Trichomycterus* sp., Xiph=*Xiphophorus* sp.

| **Species** | **Feeding habit** | **Reference** | **Sites** | | | | | |
| --- | --- | --- | --- | --- | --- | --- | --- | --- |
|  |  |  | **urban** | | | **non-urban** | | |
|  |  |  | ANC | FLOR | WPS | CAML | TNG | UBA |
| *Asty* | omnivore | Vilella *et al.* (2002) |  |  |  | x | x | x |
| *Call* | detritivore/invertivore | Lopes *et al.* (2016) |  |  | x |  |  |  |
| *Char* | invertivore | Mazzoni *et al.* (2012) |  |  |  |  | x | x |
| *Geop* | omnivore | Bastos *et al.* (2011) | x | x | x |  | x |  |
| *Gymn* | invertivore | Bonato *et al.* (2012) | x |  |  |  |  |  |
| *Hopl* | piscivore | Corrêa *et al.* (2012) |  |  |  |  | x | x |
| *Hypo* | herbivore | Delariva & Agostinho (2001) |  |  | x |  | x | x |
| *Mima* | invertivore | Wolff, Carniatto & Hahn (2013) |  |  |  |  |  | x |
| *Paro* | herbivore | Leitão *et al.* (2015) |  |  |  |  |  | x |
| *Phal* | detritivore/invertivore | Neves, Delariva & Wolff (2015) | x |  |  |  |  |  |
| *Pime* | invertivore | Rezende *et al.* (2013) |  |  |  |  | x | x |
| *Rham* | piscivore/invertivore | Bonato *et al. (*2012) | x | x | x | x | x | x |
| *Scle* | invertivore | Gonçalves, Braga & Casatti (2018) |  |  | x |  |  |  |
| *Synb* | invertivore | Teresa & Casatti (2012) |  | x |  |  | x |  |
| *Tric* | invertivore | Chara *et al.* (2006) | x |  |  |  |  |  |
| *Xiph* | omnivore | Maddern, Gill & Morgan (2011) |  | x |  |  |  |  |

Table S2. Environmental variables used in the PCA between urban stream reaches (ANC=Antes Comu, CAR=Carioca, CATO=Catonho, ELSU=Eldo sujo, FLOR=Floresta ,WPS= Water Planet sujo) and non-urban stream reaches (CAML=Camorim limpo, ELLI=Eldo limpo, JOA=Joana, TNG=Tingui, UBA=Ubatiba, WPL=Water Planet limpo). Where, URB=urbanization, BIO=fish biodiversity (guppy only, guppy+other fish), CON=specific conductivity (Spec µS/cm), TEM=temperature (^o^C), DO= Dissolved oxygen (mg/L), CA=canopy cover (%), FC= fecal coliforms (E. *coli*, MPN/100mL), NH_4_= ammonium concentration (µg/L) and sampling year (YR). Values represent averages only when followed by brackets with standard error (3-4 replicates). CON (±0.1µS/cm), TEM (±0.1^o^C) and DO (±0.1 mg/L) where measured using a multiparametric probe (YSI®) in 3 transects 10 meters apart within each stream reach. The same transects were used for estimating CA (±1%). CA was estimated using the reflection of the canopy cover on a gridded spherical convex densiometer. At each transect, four readings of % canopy cover were taken with the densiometer facing N, S, E and W then the readings were averaged to estimate CA. FC was estimated using a dilution method and NH_4_ was estimated using a fluorometric method (details in the text).

| Reach | Origin | YR | BIO | pH | CON | TEM | DO | CA | FC | NH4 |
| --- | --- | --- | --- | --- | --- | --- | --- | --- | --- | --- |
| ANC | urban | 2016 | guppy+other fish | 6.4 | 179.9 | 23.1 | 6.9 | 57.2(8.6) | 913900 | 897.7(9.4) |
| ANC | urban | 2017 | guppy+other fish | 5.6(0.03) | 113.1(1.2) | 24.5(0.05) | 3.9(0.35) | 58.8(10.8) | 75300 | 425.5(13.7) |
| CAR | urban | 2016 | guppy only | 6.2 | 518 | 24.9 | 6 | 99(0.1) | 217871.3(111805)* | 2760.5(13.6) |
| CAR | urban | 2017 | guppy only | 7.1(0.13) | 482(6.7) | 26.9(0.11) | 1.4(0.39) | 97.6(1.6) | 280900 | 12829.8(59.4) |
| CATO | urban | 2017 | guppy only | 7.2(0.02) | 369.4(14.9) | 27.5(0.17) | 3.2(0.01) | 71.5(4) | 43660 | 3399.2(2370.1)* |
| ELSU | urban | 2016 | guppy only | 7.1 | 736 | 25.4 | 4.4 | 96.8(1.1) | 217871.3(111805)* | 2548.5(61.3) |
| ELSU | urban | 2017 | guppy only | 6.2(0.07) | 251.3(13.1) | 27.5(1) | 1.2(0.4) | 95.6(2.3) | 5210 | 583(25.9) |
| FLOR | urban | 2016 | guppy+other fish | 6.4 | 361.5 | 27.2 | 6 | 84.5(3.9) | 24200 | 1655(103.5) |
| WPS | urban | 2016 | guppy+other fish | 5.9 | 354 | 25 | 4.2 | 62.4(13.4) | 3000 | 2699.1(23.4) |
| WPS | urban | 2017 | guppy+other fish | 7.1(0.08) | 462.9(7.4) | 28.3(0.09) | 3.7(0.24) | 79.4(11.6) | 396800 | 1512.1(9.3) |
| CAML | non-urban | 2016 | guppy+other fish | 6.4 | 179.8 | 22.7 | 8.3 | 94.8(0.9) | 6700 | 7.8(2.3) |
| CAML | non-urban | 2017 | guppy+other fish | 6.3(0.04) | 109.6(15.7) | 24.8(0) | 7.8(0.64) | 96.5(0.6) | 727 | 28.3(1.8) |
| ELLI | non-urban | 2016 | guppy only | 7.1 | 460 | 22.7 | 8.1 | 94.2(0.7) | 5910 | 29.4(8.2) |
| ELLI | non-urban | 2017 | guppy only | 6.2(0.11) | 188.1(0.3) | 27(0.12) | 6.5(1.2) | 98.4(0.7) | 18.7 | 10.3(0.6) |
| JOA | non-urban | 2016 | guppy only | 5.8 | 234.7 | 22.1 | 8.1(0.1)* | 95(1.8) | 256 | 14.3(4.4) |
| TNG | non-urban | 2017 | guppy+other fish | 7.2(0.18) | 108.4(0.9) | 28.5(0.04) | 7.3(1.29) | 95.8(0.6) | 882 | 9.2(1) |
| UBA | non-urban | 2017 | guppy+other fish | 5.8(0.05) | 79.2(2.5) | 28.3(0.09) | 7.4(0.4) | 92.8(1.4) | 134 | 11.7(0.8) |
| WPL | non-urban | 2016 | guppy only | 5.6 | 105.5 | 22.4 | 7.9 | 85.8(4.1) | 1460 | 7.6(6) |
| WPL | non-urban | 2017 | guppy only | 5.9(0.12) | 62.9(0.1) | 25.9(0.05) | 12.6(0.1) | 90.8(3) | 125 | 22(3.3) |

*Due to missing data, we used the mean value estimated from all reaches with the same origin in the same year. These values were only used in the PCA.

Table S3. Principal components analysis (PCA) showing the eigenvalues (Eig), variance (Var) and cumulative variance (C.var) that each principal component (PC1-8) explain. The contribution of each environmental variable to each component is also shown where CON=Specific conductivity (Spec µS/cm), TEM=Temperature (^o^C), DO= Dissolved oxygen (mg/L), CA=canopy cover (%), FC= fecal coliforms (E. *coli*, MPN/100mL), NH_4_= Ammonium concentration (µg/L) and sampling year (YR).

| Principal components | | | |  | Contribution of each variable (%) | | | | | | | |
| --- | --- | --- | --- | --- | --- | --- | --- | --- | --- | --- | --- | --- |
|  | Eig | Var (%) | C.var (%) |  | Ph | CON | TEM | DO | CA | NH4 | FC | YR |
| PC1 | 2.7 | 33.494 | 33.4944 |  | 20.4 | 16.6 | 13.5 | 21.9 | 0.07 | 21.8 | 1.71 | 4.02 |
| PC2 | 1.8 | 23.044 | 56.5381 |  | 0.53 | 14.8 | 24.2 | 0 | 4.77 | 2.08 | 15.4 | 38.3 |
| PC3 | 1.5 | 18.257 | 74.7955 |  | 5.29 | 10.6 | 0.8 | 5.92 | 50.2 | 0 | 21.3 | 5.96 |
| PC4 | 0.8 | 9.5545 | 84.3499 |  | 26.2 | 1.64 | 2.14 | 28.5 | 2.32 | 3.67 | 33.9 | 1.69 |
| PC5 | 0.6 | 7.1756 | 91.5255 |  | 12 | 6.91 | 4.24 | 2.21 | 14.4 | 50.8 | 6.92 | 2.46 |
| PC6 | 0.3 | 3.8256 | 95.3512 |  | 27.2 | 6.46 | 20.7 | 2.89 | 13.3 | 8.65 | 16.7 | 4.11 |
| PC7 | 0.2 | 3.1218 | 98.4729 |  | 2.71 | 3.23 | 26.7 | 32 | 13.9 | 11.8 | 4.03 | 5.73 |
| PC8 | 0.1 | 1.5271 | 100 |  | 5.68 | 39.8 | 7.71 | 6.62 | 1.02 | 1.24 | 0.12 | 37.8 |

Table S4. Generalized linear models (GLMMs) testing for differences in environmental and guppy population variables between urban and non-urban sites. Separate models were built for each of the response variables: NH_4_=concentration of ammonium per reach (µg/L), FC=concentration of fecal coliforms (E. *coli*) per reach (MPN/100mL), GD=estimated guppy density (ind/ m^2^), IB= biomass of all invertebrates taxa per reach (mg/m^2^), CB=biomass of chironomids per reach (mg/m^2^). Where UR= urbanization (urban or non-urban), YR=sampling year, BIO=fish biodiversity (guppy only and guppy with other fish) are fixed effects. The variance explained by both fixed plus random factor (conditional R square, R^2^_c_), and the variance explained only by the fixed factors (marginal R square, R^2^_m_) are shown. SE represent the standard error and the collinearity test was based on variance inflation ratios (VIF).

| Response variable | Model family | R^2^_m_ | R^2^_c_ | (n) | Fixed effects | Estimate | SE | t/z value | p-value | VIF |
| --- | --- | --- | --- | --- | --- | --- | --- | --- | --- | --- |
| NH4 |  |  |  |  |  |  |  |  |  |  |
|  | Gamma (log link) | 0.96 | 0.96 | 18 | UR | 4.27 | 0.34 | 12.5 | <0.001 | 1.74 |
|  |  |  |  |  | YR | -0.26 | 0.28 | -0.93 | 0.35 | 1.35 |
|  |  |  |  |  | BIO | 0.03 | 0.31 | 0.12 | 0.9 | 1.20 |
|  |  |  |  |  | GD | 0.59 | 0.17 | 3.46 | <0.001 | 1.70 |
|  |  |  |  |  | int | 3.14 | 0.39 | 3.46 | <0.001 |  |
| FC |  |  |  |  |  |  |  |  |  |  |
|  | Gamma (log link) | 0.89 | 0.89 | 17 | UR | 5.46 | 0.70 | 7.8 | <0.001 | 1.30 |
|  |  |  |  |  | YR | -1.66 | 0.72 | -2.31 | 0.02 | 1.34 |
|  |  |  |  |  | BIO | -0.40 | 0.63 | -0.63 | 0.53 | 1.03 |
|  |  |  |  |  | int | 8.06 | 0.59 | 13.72 | <0.001 |  |
| GD |  |  |  |  |  |  |  |  |  |  |
|  | Gamma (log link) | 0.84 | 0.92 | 19 | UR | 3.11 | 0.46 | 6.75 | <0.001 | 1.01 |
|  |  |  |  |  | YR | 0.53 | 0.22 | 2.37 | 0.02 | 1.01 |
|  |  |  |  |  | BIO | 0.64 | 0.46 | 1.39 | 0.16 | 1.00 |
|  |  |  |  |  | int | 0.15 | 0.42 | 0.36 | 0.72 |  |
| IB |  |  |  |  |  |  |  |  |  |  |
|  | Gamma (log link) | 0.48 | 0.48 | 19 | UR | 1.39 | 0.48 | 2.90 | <0.01 | 1.60 |
|  |  |  |  |  | YR | -0.29 | 0.41 | -0.71 | 0.48 | 1.17 |
|  |  |  |  |  | BIO | -0.43 | 0.42 | -1.02 | 0.31 | 1.25 |
|  |  |  |  |  | GD | -0.004 | 0.004 | -1.17 | 0.24 | 1.65 |
|  |  |  |  |  | int | 4.01 | 0.44 | 9.08 | <0.001 |  |
| CB |  |  |  |  |  |  |  |  |  |  |
|  | Gamma (log link) | 0.57 | 0.57 | 19 | UR | 1.97 | 0.53 | 3.69 | <0.001 | 1.60 |
|  |  |  |  |  | YR | -0.42 | 0.46 | -0.90 | 0.37 | 1.20 |
|  |  |  |  |  | BIO | -0.06 | 0.47 | -0.12 | 0.90 | 1.24 |
|  |  |  |  |  | GD | -0.01 | 0.00 | -1.38 | 0.17 | 1.64 |
|  |  |  |  |  | int | 3.26 | 0.52 | 6.32 | <0.001 |  |

Table S5. Guppy population density and life history traits in urban stream reaches (ANC=Antes Comu, CAR=Carioca, CATO=Catonho, ELSU=Eldo sujo, FLOR=Floresta ,WPS= Water Planet sujo) and non-urban stream reaches (CAML=Camorim limpo, ELLI=Eldo limpo, JOA=Joana, TNG=Tingui, UBA=Ubatiba, WPL=Water Planet limpo). Data is shown for each sampling year (YR). Where, BIO=fish biodiversity (guppy only and guppy with other fish), GD= estimated guppy density (ind/m^2^), N= sample size for estimating number of offspring, condition, gonad weight, and reproductive allotment, NO= average number of offspring, CO= average condition (hepatosomatic index), GW=average gonad weight (mg), RA=average reproductive allotment (gonad dry weight / total body dry weight minus gonads, mg).

| Reach | Origin | YR | BIO | GD (se) | N | NO (se) | CO (se) | GW (se) | RA (se) |
| --- | --- | --- | --- | --- | --- | --- | --- | --- | --- |
| ANC | urban | 2016 | guppy+other fish | 49.4(160) | 36 | 15.1(2.1) | 4.4(0.5) | 13.7(2.6) | 0.14(0.02) |
| ANC | urban | 2017 | guppy+other fish | 10.6(9) | 45 | 25.6(2.9) | 3.1(0.2) | 28(4.2) | 0.21(0.02) |
| CAR | urban | 2016 | guppy only | 151.6(0.5) | 61 | 13.4(1.2) | 3.8(0.2) | 17.6(2.1) | 0.16(0.01) |
| CAR | urban | 2017 | guppy only | 367.3(0.3) | 47 | 14.6(1.9) | 4.1(0.2) | 11.8(2.2) | 0.1(0.01) |
| CATO | urban | 2017 | guppy only | 47.6(1) | 48 | 20.5(1.9) | 3.6(0.2) | 17.4(2.4) | 0.2(0.02) |
| FLOR | urban | 2016 | guppy+other fish | 37.7(0.4) | 45 | 6.8(0.4) | 2.4(0.1) | 6.8(0.7) | 0.14(0.01) |
| ELSU | urban | 2016 | guppy only | 24.7(1) | 58 | 9.9(0.7) | 5(0.2) | 8.2(0.7) | 0.2(0.01) |
| ELSU | urban | 2017 | guppy only | 44.9(0.1) | 41 | 8.2(1) | 2(0.1) | 9.1(1.3) | 0.14(0.02) |
| WPS | urban | 2016 | guppy+other fish | 22.3(4) | 50 | 11.1(0.8) | 3(0.1) | 11.1(1) | 0.19(0.02) |
| WPS | urban | 2017 | guppy+other fish | 28.4(2) | 63 | 9.2(0.9) | 2.6(0.2) | 8.9(1.1) | 0.16(0.01) |
| CAML | non-urban | 2016 | guppy+other fish | 0.6(17) | 36 | 8.6(1.1) | 1.8(0.1) | 10.8(1.2) | 0.15(0.01) |
| CAML | non-urban | 2017 | guppy+other fish | 2.3(16) | 9 | 4.4(0.6) | 1.2(0.3) | 4.8(0.8) | 0.15(0.02) |
| ELLI | non-urban | 2016 | guppy only | 2.6(4) | 54 | 3.7(0.4) | 2.6(0.2) | 3.8(0.5) | 0.14(0.01) |
| ELLI | non-urban | 2017 | guppy only | 7.5(0.4) | 37 | 4.5(0.4) | 1.6(0.1) | 4.7(0.5) | 0.13(0.01) |
| JOA | non-urban | 2016 | guppy only | 0.9(13) | 12 | 13.8(2) | 2.5(0.3) | 12.8(2.4) | 0.24(0.03) |
| TNG | non-urban | 2017 | guppy+other fish | 3.6(10) | 45 | 15.6(1.6) | 1.9(0.1) | 14.3(2.1) | 0.14(0.01) |
| UBA | non-urban | 2017 | guppy+other fish | 1.2(16) | 8 | 14.1(3.5) | 1.3(0.2) | 8.8(2.2) | 0.15(0.03) |
| WPL | non-urban | 2016 | guppy only | 4.1(13) | 18 | 5.6(0.8) | 2.7(0.5) | 8.5(1.4) | 0.17(0.02) |
| WPL | non-urban | 2017 | guppy only | 7.2(109) | 22 | 10(1.8) | 2.1(0.2) | 12(1.9) | 0.18(0.02) |

Table S6. Generalized linear models (GLMMs) testing for differences in guppy diets and traits between urban and non-urban sites. Separate models were built for each of the response variables: NO=number of offspring per female, CO=female condition (HSI), PC=proportion of chironomids consumed, PO= proportion of other invertebrates consumed. Where UR= urbanization (urban or non-urban), YR=sampling year, BIO=fish biodiversity (guppy only and guppy with other fish), SL= standard guppy length (mm) and GD=guppy density (ind/m^2^) are the fixed effects. SE represents the standard error. The variance explained by both fixed plus random factor (conditional R square, R^2^_c_), and the variance explained only by the fixed factors (marginal R square, R^2^_m_) is shown. SE represent the standard error and the collinearity test was based on variance inflation ratios (VIF).

| Response variable | Model family | R^2^_m_ | R^2^_c_ | (n) | Fixed effects | Estimate | SE | t/z value | p-value | VIF |
| --- | --- | --- | --- | --- | --- | --- | --- | --- | --- | --- |
| NO | Poisson | 0.63 | 0.81 | 735 | UR | 0.35 | 0.17 | 2.10 | 0.04 | 1.01 |
|  |  |  |  |  | YR | 0.01 | 0.03 | 0.48 | 0.63 | 1.24 |
|  |  |  |  |  | BIO | 0.06 | 0.17 | 0.33 | 0.74 | 1 |
|  |  |  |  |  | SL | 0.53 | 0.01 | 49.85 | <0.001 | 1.04 |
|  |  |  |  |  | GD | -0.14 | 0.02 | -6.02 | <0.001 | 1.24 |
|  |  |  |  |  | int | 1.98 | 0.15 | 13.27 | <0.001 |  |
|  |  |  |  |  |  |  |  |  |  |  |
| CO | Gamma (log link) | 0.39 | 0.44 | 735 | UR | 0.40 | 0.15 | 2.70 | <0.01 | 1.03 |
|  |  |  |  |  | YR | -0.39 | 0.04 | -9.37 | <0.001 | 1.27 |
|  |  |  |  |  | BIO | 0.22 | 0.14 | 1.49 | 0.14 | 1.01 |
|  |  |  |  |  | SL | -0.03 | 0.02 | -1.68 | 0.09 | 1.02 |
|  |  |  |  |  | GD | 0.16 | 0.04 | 4.38 | <0.01 | 1.30 |
|  |  |  |  |  | int | 0.84 | 0.13 | 6.48 | <0.001 |  |
|  |  |  |  |  |  |  |  |  |  |  |
| PC | Binomial | 0.55 | 0.62 | 230 | UR | 3.94 | 1.04 | 3.78 | <0.001 | 1.23 |
|  |  |  |  |  | YR | -0.49 | 0.54 | -0.91 | 0.37 | 1.07 |
|  |  |  |  |  | BIO | 1.47 | 0.83 | 1.77 | 0.08 | 1.29 |
|  |  |  |  |  | SL | -0.20 | 0.25 | -0.78 | 0.43 | 1.07 |
|  |  |  |  |  | GD | -1.01 | 0.36 | -2.79 | 0.01 | 1.29 |
|  |  |  |  |  | int | -5.46 | 1.15 | -4.74 | <0.001 |  |
|  |  |  |  |  |  |  |  |  |  |  |
| PO | Binomial | 0.17 | 0.26 | 230 | UR | -1.10 | 0.57 | -1.92 | 0.05 | 1.05 |
|  |  |  |  |  | YR | -1.17 | 0.45 | -2.59 | <0.01 | 1.33 |
|  |  |  |  |  | BIO | -0.09 | 0.61 | -0.15 | 0.88 | 1.22 |
|  |  |  |  |  | SL | -0.30 | 0.22 | -1.34 | 0.18 | 1.19 |
|  |  |  |  |  | GD | -0.18 | 0.26 | -0.67 | 0.50 | 1.27 |
|  |  |  |  |  | int | -0.18 | 0.59 | -0.74 | 0.46 |  |

Table S7. LMM models testing the relationship between guppy traits and diet. We built separate models using body length (SL, mm), number of offspring (NO) and guppy condition (CO, I) as response variables and sampling year (YR), fish biodiversity (guppy only and guppy and other fish, BIO), body length (SL, mm), guppy density (GD, ind/m^2^) and the proportion of chironomids consumed (PC) as fixed factors. We used reach identity as a random factor. Following model selection, the coefficients of the best models (∆AICc <2) were estimated and the averaged coefficients are shown. The R^2^_c_ and R^2^_m_ show the range of conditional and marginal R^2^ values for the best models. A table with the full model selection showing all the candidate models can be found in the appendix (Table S8).

|  |  | Averaged coefficients | | | |  | |  | |  | |
| --- | --- | --- | --- | --- | --- | --- | --- | --- | --- | --- | --- |
| Response variable | adjR^2^ | YR | BIO | SL | GD | | PC | | (Int) | |  |
| NO | 77-82 | 1.18 | -0.66 | 1.58 | – | | 19.48 | | -28.76 | |  |
| CO | 26-36 | -0.19 |  | – | – | | 3.35 | | 2.43 | |  |

(-) indicates a factor that was not included in the model

Table S8. Full model selection with all candidate LMM models (C.model) testing the relationship between guppy traits and diet. We built separate models using number of offspring (NO) and guppy condition (CO) as response variables and sampling year (YR), fish biodiversity (guppy only and guppy and other fish, BIO), body length (SL, mm), guppy density (GD, ind/m^2^) and the proportion of chironomid consumed (PC, %) as fixed factors. We used reach identity as random factor. Following model selection, the coefficients of the best models (∆AICc <2) were estimated and averages were calculated when necessary. AICc show the corrected Akaike information criterion values, while ∆AICc shows the difference in AICc between the current and the most appropriate model, and weight show the Akaike weights. For each model we calculated the adjusted R^2^ (adjR^2^) which takes into account only the variation explained by the factors that affect the response variable and the weight of evidence in favor of each model (W).

| Fixed factor | C.model | df | logLik | AICc | ∆AICc | adjR^2 | W | YR | BIO | SL | GD | PC | (Int) |
| --- | --- | --- | --- | --- | --- | --- | --- | --- | --- | --- | --- | --- | --- |
| NO | 1 | 6 | -40.40 | 99.8 | 0.00 | 0.81 | 0.35 | + | - | 1.50 | - | 20.26 | -27.67 |
|  | 2 | 5 | -42.96 | 100.5 | 0.74 | 0.77 | 0.24 | - | - | 1.74 | - | 17.52 | -31.91 |
|  | 3 | 7 | -38.39 | 101.0 | 1.16 | 0.82 | 0.20 | + | + | 1.45 | - | 21.36 | -25.69 |
|  | 4 | 6 | -41.06 | 101.1 | 1.33 | 0.78 | 0.18 | - | + | 1.68 | - | 18.59 | -29.97 |
|  | 5 | 6 | -44.21 | 107.4 | 7.63 | 0.61 | 0.01 | + | + | - | - | 24.13 | 8.14 |
|  | 6 | 5 | -46.63 | 107.9 | 8.09 | 0.57 | 0.01 | + | - | - | - | 22.36 | 7.03 |
|  | 7 | 5 | -48.07 | 110.8 | 10.96 | 0.46 | 0.00 | - | + | - | - | 20.10 | 10.48 |
|  | 8 | 4 | -50.45 | 111.8 | 11.96 | 0.42 | 0.00 | - | - | - | - | 18.04 | 9.39 |
|  | 9 | 6 | -46.62 | 112.2 | 12.44 | 0.77 | 0.00 | - | - | 1.79 | -0.004 | 17.52 | -33.04 |
|  | 10 | 7 | -44.06 | 112.3 | 12.50 | 0.81 | 0.00 | + | - | 1.56 | -0.01 | 20.04 | -28.96 |
|  | 11 | 4 | -50.85 | 112.5 | 12.75 | 0.56 | 0.00 | - | - | 1.93 | - | - | -34.42 |
|  | 12 | 5 | -49.19 | 113.0 | 13.20 | 0.56 | 0.00 | - | + | 1.89 | - | - | -33.48 |
|  | 13 | 5 | -49.37 | 113.4 | 13.56 | 0.56 | 0.00 | + | - | 1.93 | - | - | -34.66 |
|  | 14 | 7 | -44.76 | 113.7 | 13.90 | 0.78 | 0.00 | - | + | 1.70 | -0.002 | 18.51 | -30.41 |
|  | 15 | 6 | -47.70 | 114.4 | 14.61 | 0.56 | 0.00 | + | + | 1.90 | - | - | -33.72 |
|  | 16 | 8 | -42.13 | 114.7 | 14.86 | 0.82 | 0.00 | + | + | 1.47 | -0.002 | 21.21 | -26.24 |
|  | 17 | 7 | -47.41 | 119.0 | 19.21 | 0.62 | 0.00 | + | + | - | 0.01 | 24.41 | 8.11 |
|  | 18 | 6 | -50.02 | 119.0 | 19.25 | 0.58 | 0.00 | + | - | - | 0.01 | 22.45 | 6.91 |
|  | 19 | 5 | -52.27 | 119.2 | 19.36 | 0.23 | 0.00 | + | + | - | - | - | 11.16 |
|  | 20 | 4 | -54.16 | 119.2 | 19.38 | 0.19 | 0.00 | - | + | - | - | - | 12.09 |
|  | 21 | 4 | -54.36 | 119.6 | 19.79 | 0.23 | 0.00 | + | - | - | - | - | 10.70 |
|  | 22 | 3 | -56.28 | 120.2 | 20.36 | 0.19 | 0.00 | - | - | - | - | - | 11.62 |
|  | 23 | 6 | -50.95 | 120.9 | 21.11 | 0.49 | 0.00 | - | + | - | 0.01 | 20.44 | 10.23 |
|  | 24 | 5 | -53.59 | 121.8 | 21.99 | 0.44 | 0.00 | - | - | - | 0.01 | 18.19 | 9.01 |
|  | 25 | 5 | -54.28 | 123.2 | 23.37 | 0.56 | 0.00 | - | - | 1.94 | 0.00 | - | -34.52 |
|  | 26 | 6 | -52.53 | 124.1 | 24.26 | 0.56 | 0.00 | - | + | 1.92 | -0.01 | - | -33.98 |
|  | 27 | 6 | -52.79 | 124.6 | 24.78 | 0.56 | 0.00 | + | - | 1.94 | -0.004 | - | -34.62 |
|  | 28 | 7 | -51.02 | 126.2 | 26.43 | 0.56 | 0.00 | + | + | 1.91 | -0.005 | - | -34.02 |
|  | 29 | 5 | -57.33 | 129.3 | 29.47 | 0.19 | 0.00 | - | + | - | 0.004 | - | 12.02 |
|  | 30 | 5 | -57.57 | 129.8 | 29.95 | 0.23 | 0.00 | + | - | - | 0.0005 | - | 10.68 |
|  | 31 | 6 | -55.42 | 129.8 | 30.04 | 0.23 | 0.00 | + | + | - | 0.001 | - | 11.15 |
|  | 32 | 4 | -59.50 | 129.9 | 30.06 | 0.19 | 0.00 | - | - | - | 0.003 | - | 11.49 |
| CO |  |  |  |  |  |  |  |  |  |  |  |  |  |
|  | 1 | 4 | -23.96 | 58.8 | 0.00 | 0.26 | 0.40 | - | - | - | - | 3.56 | 2.31 |
|  | 2 | 5 | -22.87 | 60.4 | 1.57 | 0.36 | 0.18 | + | - | - | - | 2.89 | 2.70 |
|  | 3 | 4 | -25.27 | 61.4 | 2.62 | 0.26 | 0.11 | + | - | - | - | - | 3.14 |
|  | 4 | 5 | -23.50 | 61.6 | 2.84 | 0.30 | 0.10 | - | + | - | - | 3.30 | 2.14 |
|  | 5 | 3 | -27.55 | 62.7 | 3.93 | 0.02 | 0.06 | - | - | - | - | - | 2.70 |
|  | 6 | 5 | -24.40 | 63.4 | 4.63 | 0.32 | 0.04 | + | + | - | - | - | 2.83 |
|  | 7 | 6 | -22.38 | 63.8 | 4.98 | 0.39 | 0.03 | + | + | - | - | 2.39 | 2.55 |
|  | 8 | 4 | -26.63 | 64.1 | 5.33 | 0.10 | 0.03 | - | + | - | - | - | 2.39 |
|  | 9 | 5 | -25.15 | 64.9 | 6.13 | 0.27 | 0.02 | - | - | 0.04 | - | 3.43 | 1.28 |
|  | 10 | 6 | -23.66 | 66.3 | 7.54 | 0.40 | 0.01 | + | - | 0.11 | - | 2.44 | 0.24 |
|  | 11 | 5 | -25.88 | 66.4 | 7.58 | 0.32 | 0.01 | + | - | 0.13 | - | - | 0.01 |
|  | 12 | 4 | -28.55 | 68.0 | 9.17 | 0.05 | 0.00 | - | - | 0.07 | - | - | 0.93 |
|  | 13 | 6 | -24.58 | 68.2 | 9.37 | 0.31 | 0.00 | - | + | 0.07 | - | 3.08 | 0.57 |
|  | 14 | 6 | -24.83 | 68.7 | 9.87 | 0.39 | 0.00 | + | + | 0.15 | - | - | -0.77 |
|  | 15 | 5 | -27.45 | 69.5 | 10.73 | 0.40 | 0.00 | - | - | - | 0.005 | 2.94 | 2.18 |
|  | 16 | 5 | -27.52 | 69.6 | 10.87 | 0.13 | 0.00 | - | + | 0.10 | - | - | 0.07 |
|  | 17 | 6 | -25.43 | 69.9 | 11.07 | 0.55 | 0.00 | + | - | - | 0.01 | 2.01 | 2.65 |
|  | 18 | 5 | -27.66 | 69.9 | 11.15 | 0.48 | 0.00 | + | - | - | 0.01 | - | 2.96 |
|  | 19 | 7 | -22.95 | 70.1 | 11.31 | 0.44 | 0.00 | + | + | 0.13 | - | 1.89 | -0.53 |
|  | 20 | 4 | -30.66 | 72.2 | 13.39 | 0.23 | 0.00 | - | - | - | 0.01 | - | 2.48 |
|  | 21 | 6 | -27.26 | 73.5 | 14.74 | 0.41 | 0.00 | - | + | - | 0.004 | 2.82 | 2.10 |
|  | 22 | 6 | -27.30 | 73.6 | 14.81 | 0.49 | 0.00 | + | + | - | 0.01 | - | 2.82 |
|  | 23 | 7 | -25.27 | 74.7 | 15.94 | 0.55 | 0.00 | + | + | - | 0.01 | 1.85 | 2.60 |
|  | 24 | 5 | -30.25 | 75.1 | 16.34 | 0.25 | 0.00 | - | + | - | 0.005 | - | 2.32 |
|  | 25 | 6 | -28.68 | 76.4 | 17.58 | 0.50 | 0.00 | + | - | 0.08 | 0.01 | - | 1.03 |
|  | 26 | 6 | -28.74 | 76.5 | 17.70 | 0.40 | 0.00 | - | - | -0.003 | 0.005 | 2.93 | 2.26 |
|  | 27 | 7 | -26.59 | 77.4 | 18.59 | 0.56 | 0.00 | + | - | 0.06 | 0.01 | 1.86 | 1.29 |
|  | 28 | 5 | -31.85 | 78.3 | 19.53 | 0.23 | 0.00 | - | - | 0.03 | 0.01 | - | 1.79 |
|  | 29 | 7 | -28.20 | 80.6 | 21.81 | 0.52 | 0.00 | + | + | 0.10 | 0.01 | - | 0.51 |
|  | 30 | 7 | -28.48 | 81.1 | 22.36 | 0.41 | 0.00 | - | + | 0.01 | 0.004 | 2.79 | 1.89 |
|  | 31 | 6 | -31.34 | 81.7 | 22.91 | 0.26 | 0.00 | - | + | 0.05 | 0.005 | - | 1.20 |
|  | 32 | 8 | -26.32 | 83.0 | 24.26 | 0.57 | 0.00 | + | + | 0.07 | 0.01 | 1.65 | 0.88 |
| SL |  |  |  |  |  |  |  |  |  |  |  |  |  |
|  | 1 | 4 | -36.76 | 84.4 | 0.00 | 0.09 | 0.23 | - | - | NA | - | 1.13 | 23.66 |
|  | 2 | 5 | -35.12 | 84.9 | 0.47 | 0.19 | 0.18 | + | - | NA | - | 2.78 | 22.83 |
|  | 3 | 3 | -38.84 | 85.3 | 0.90 | 0.08 | 0.15 | - | - | NA | - | - | 23.81 |
|  | 4 | 5 | -35.56 | 85.7 | 1.36 | 0.11 | 0.12 | - | + | NA | - | 1.53 | 23.95 |
|  | 5 | 4 | -37.50 | 85.9 | 1.47 | 0.15 | 0.11 | + | - | NA | - | - | 23.31 |
|  | 6 | 4 | -37.71 | 86.3 | 1.89 | 0.09 | 0.09 | - | + | NA | - | - | 24.09 |
|  | 7 | 6 | -33.93 | 86.9 | 2.48 | 0.21 | 0.07 | + | + | NA | - | 3.22 | 23.15 |
|  | 8 | 5 | -36.42 | 87.4 | 3.06 | 0.17 | 0.05 | + | + | NA | - | - | 23.59 |
|  | 9 | 5 | -40.34 | 95.3 | 10.91 | 0.15 | 0.00 | - | - | NA | 0.01 | 0.99 | 23.41 |
|  | 10 | 4 | -42.41 | 95.7 | 11.29 | 0.15 | 0.00 | - | - | NA | 0.01 | - | 23.54 |
|  | 11 | 5 | -41.05 | 96.7 | 12.33 | 0.18 | 0.00 | - | + | NA | 0.01 | - | 23.96 |
|  | 12 | 6 | -38.86 | 96.7 | 12.34 | 0.20 | 0.00 | - | + | NA | 0.01 | 1.69 | 23.81 |
|  | 13 | 6 | -38.95 | 96.9 | 12.52 | 0.23 | 0.00 | + | - | NA | 0.01 | 2.33 | 22.76 |
|  | 14 | 5 | -41.27 | 97.2 | 12.77 | 0.20 | 0.00 | + | - | NA | 0.01 | - | 23.16 |
|  | 15 | 6 | -39.98 | 99.0 | 14.58 | 0.23 | 0.00 | + | + | NA | 0.01 | - | 23.56 |
|  | 16 | 7 | -37.51 | 99.2 | 14.82 | 0.27 | 0.00 | + | + | NA | 0.01 | 2.91 | 23.18 |

(-) indicates a factor not included in the model

(+) indicates a categorical factor included in the model

Figure S1. Map showing (a) Brazil and (b) the state of Rio de Janeiro, where (c) 12 stream reaches were studied. The red placemarks show the location of each urban stream reach (ANC=Antes Comu, CAR=Carioca, CATO=Catonho, ELSU=Eldo sujo, FLOR=Floresta, WPS= Water Planet sujo). While the blue placemarks show the non-urban stream reaches (CAML=Camorim limpo, ELLI=Eldo limpo, JOA=Joana, TNG=Tingui, UBA=Ubatiba, WPL=Water Planet limpo). Maps obtained from Google earth V7.3.3.7699 (December 30^th^, 2016). Rio de Janeiro, RJ, Brazil. Copyright: Google 2020. Accessed on May 10, 2020. Map (b) coordinates: 22^o^44`19.26”S 41^o^56`37.50”W, eye alt 554.90km. Map (c) coordinates: 22^o^54`54.88”S 43^o^04`14.94W, eye alt 89.27km. Urban stream reach coordinates: ANC(22°58'23.40"S,43°17'1.43"W), CAR(22°56'22.43"S,43°12'5.06"W),CATO(22°54'18.88"S,43°22'47.89"W),ELSU(22°56'30.66"S,43°19'25.21"W),FLOR(22°55'45.67"S,43°19'16.80"W),WPS(22°59'5.44"S,43°29'22.67"W). Non-urban stream reach coordinates: CAML(22°57'55.93"S,43°25'6.49"W),ELLI(22°56'29.28"S,43°19'22.27"W),JOA(22°55'45.75"S,43°16'4.85"W),TNG(22°50'14.95"S,42°36'25.57"W),UBA(22°52'15.81"S,42°44'14.15"W), WPL(22°57'44.67"S, 43°28'26.64"W).


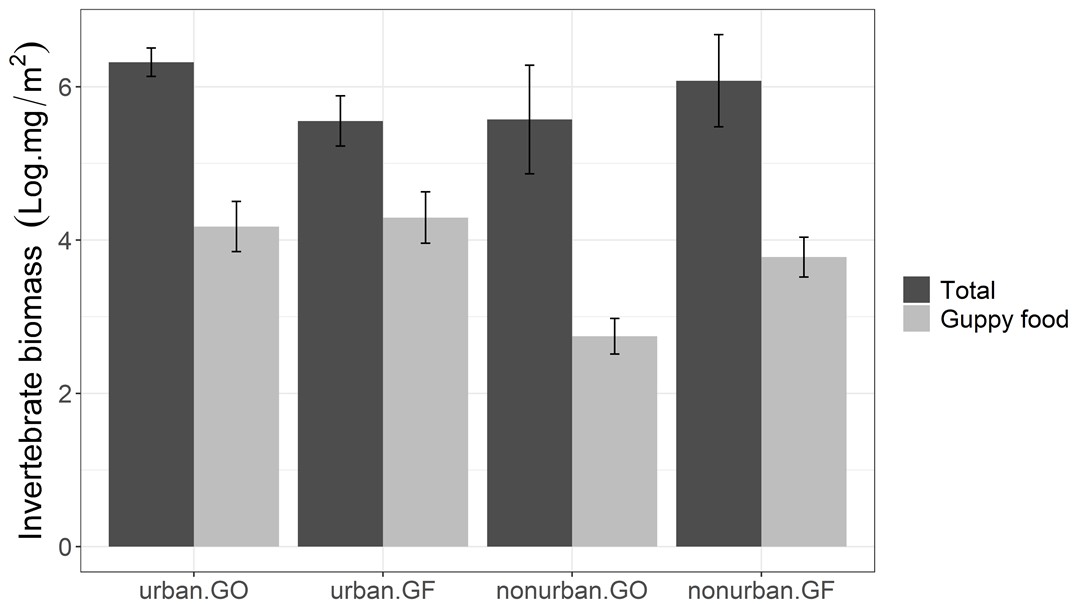


Figure S2. Total invertebrate biomass (dark grey) and invertebrate biomass available as food for guppies (light grey) found in urban and non-urban reaches. Biomass was estimated as dry mass of invertebrate per reach area (Log mg/m^2^). Bars indicate the mean and lines are the standard error of the mean. Data shown includes both sampling years 2016 and 2017.

Figure S3. Guppy life history traits. Gonad weight (panel a) and reproductive allotment (panel b) of urban and non-urban guppies. In both conditions, guppies occur in stream reaches with other fish species (black circles, guppy+other fish) and in reaches where guppies are the only fish species (grey triangle, guppy only). Symbols represent the mean for all the reaches, while bars are the standard error of the mean. Data shown includes both sampling years 2016 and 2017.

Figure S4. Proportion of each food item found in the guts of guppies in urban reaches where guppies occur with other fish species (urban:guppy+other fish), urban reaches where guppies are the only fish species (urban:guppy only), non-urban reaches where guppies occur with other fish species (nonurban:guppy+other fish) and non-urban reaches where guppies are the only fish species (nonurban:guppy only). Each shade of grey represents one food item, bars represent the mean and lines are the standard error of the mean. Data shown includes both sampling years 2016 and 2017.
